# The protective role of DOT1L in UV-induced melanomagenesis

**DOI:** 10.1038/s41467-017-02687-7

**Published:** 2018-01-17

**Authors:** Bo Zhu, Shuyang Chen, Hongshen Wang, Chengqian Yin, Changpeng Han, Cong Peng, Zhaoqian Liu, Lixin Wan, Xiaoyang Zhang, Jie Zhang, Christine G. Lian, Peilin Ma, Zhi-xiang Xu, Sharon Prince, Tao Wang, Xiumei Gao, Yujiang Shi, Dali Liu, Min Liu, Wenyi Wei, Zhi Wei, Jingxuan Pan, Yongjun Wang, Zhenyu Xuan, Jay Hess, Nicholas K. Hayward, Colin R. Goding, Xiang Chen, Jun Zhou, Rutao Cui

**Affiliations:** 1 Department of Pharmacology and Experimental Therapeutics, Boston University School of Medicine, Boston, MA 02118 USA; 2 Shandong Provincial Key Laboratory of Animal Resistance Biology, Institute of Biomedical Sciences, College of Life Sciences, Shandong Normal University, 250014 Jinan, China; 3 Department of Dermatology & China Hunan key Laboratory of Skin Cancer and Psoriasis, Xiangya Hospital, Central South University, 410008 Changsha, China; 4 Shanghai University of Traditional Chinese Medicine, 201203 Shanghai, China; 5 Institute of Life Science, Jiangsu University, 212013 Zhenjiang, China; 6 Department of Molecular Oncology, H. Lee Moffitt Cancer Center and Research Institute, Tampa, FL 33612 USA; 7 Dana-Farber Cancer Institute, 450 Brookline Avenue, Boston, MA 02215 USA; 8 Department of Computer Science, New Jersey Institute of Technology, Newark, NJ 07102 USA; 9 Department of Pathology, The Brigham and Women’s Hospital, Harvard Medical School, 221 Longwood Ave, Boston, MA 02115 USA; 10 Department of Pathology, Indiana University School of Medicine, 340 West 10th Street, Fairbanks 6200, Indianapolis, IN 46202 USA; 11 Division of Hematology/Oncology, Department of Medicine, University of Alabama at Birmingham School of Medicine, Birmingham, AL 35233 USA; 12 Department of Human Biology, University of Cape Town, Rondebosch, Cape Town 7700 South Africa; 13 Tianjin State Key Laboratory of Modern Chinese Medicine, Tianjin University of Traditional Chinese Medicine, 300193 Tianjin, China; 14 Department of Medicine, Endocrinology, Brigham and Women’s Hospital, Harvard Medical School, 75 Francis Street, Boston, MA 02115 USA; 15 Department of Chemistry and Biochemistry, Loyola University Chicago, Chicago, IL 60660 USA; 16 Department of Pathology, Beth Israel Deaconess Medical Center, Harvard Medical School, Boston, MA 02115 USA; 17 Cancer Pharmacology Research Institute, Jinan University, 510632 Guangzhou, China; 18 Department of Biological Sciences, Center for Systems Biology, University of Texas at Dallas, Richardson, TX 75080 USA; 19 QIMR Berghofer Medical Research Institute, Brisbane City, QLD 4006 Australia; 20 Ludwig Institute for Cancer Research, University of Oxford, Headington, Oxford, OX3 7DQ UK

## Abstract

The DOT1L histone H3 lysine 79 (H3K79) methyltransferase plays an oncogenic role in MLL-rearranged leukemogenesis. Here, we demonstrate that, in contrast to MLL-rearranged leukemia, DOT1L plays a protective role in ultraviolet radiation (UVR)-induced melanoma development. Specifically, the *DOT1L* gene is located in a frequently deleted region and undergoes somatic mutation in human melanoma. Specific mutations functionally compromise DOT1L methyltransferase enzyme activity leading to reduced H3K79 methylation. Importantly, in the absence of DOT1L, UVR-induced DNA damage is inefficiently repaired, so that DOT1L loss promotes melanoma development in mice after exposure to UVR. Mechanistically, DOT1L facilitates DNA damage repair, with DOT1L-methylated H3K79 involvement in binding and recruiting XPC to the DNA damage site for nucleotide excision repair (NER). This study indicates that DOT1L plays a protective role in UVR-induced melanomagenesis.

## Abstract (web summary)

The interaction of DOT1L with MLL oncogenic fusion proteins has been implicated in leukemogenesis. Here, the authors show a contrasting role for DOT1L in protecting UVR-induced melanomagenesis by facilitating DNA repair through interaction with XPC.

## Introduction

The epigenetic landscape of cells, comprising a combination of DNA methylation and hydroxymethylation together with posttranslational histone modifications, is a crucial determinant in the establishment and maintenance of gene expression programs that govern cell identity. Thus it is not surprising that disease-associated alterations in the function of histone-modifying complexes disrupt the pattern and levels of histone marks and DNA methylation and consequently deregulate the control of chromatin-based processes^1^. Importantly, deregulation of epigenetic processes can contribute to transformation and the development of cancer. For example, genome-wide mapping of 5-hydroxymethylcytosine (5-hmC), an intermediate in cytosine dimethylation mediated by the TET enzymes, revealed that loss of 5-hmC is an epigenetic hallmark of melanoma, with diagnostic and prognostic implications^2^. Histone methylation, catalyzed by a group of histone methyltransferases, takes place on both lysine (K) and arginine (R) residues^3^. Unlike other modified histone *N*-terminal “tail” residues, H3K79 is exposed on the nucleosome surface and methylated within the globular domain^4,5^. The yeast protein Dot1 and human homolog DOT1L are responsible for catalyzing H3K79 mono-, di- and tri-methylation^5,6^. DOT1L-dependent H3K79 methylation is associated with telomere silencing, meiotic checkpoint control, modulation of constitutive heterochromatin, transcriptional activation, and DNA repair^7,8^.

Nucleotide excision repair (NER) is a particularly critical process that removes DNA damage induced by ultraviolet radiation (UVR), requiring the coordinated action of about 30 proteins^9^. In mammals, NER removes UVR-induced photoproducts, including cyclobutane-pyrimidine dimers (CPDs) and 6–4 pyrimidine-pyrimidone photoproducts (6–4 PPs). The xeroderma pigmentosum, complementation group C (XPC) complex, containing RAD23B and centrin 2, recognizes DNA lesions and initiates global genome NER. However, as CPDs do not create helix distortions that can be directly recognized by XPC, the DDB1/DDB2 complex is required to stabilize DNA conformation and assist XPC binding after UVR^10^. After the initial recognition step, repair proteins, such as XPA, and TFIIH complex including the essential subunit p62, bind to the damage site, which promotes recruitment of other NER factors. This allows incision of the damaged strand by repair endonucleases, including ERCC1/XPF and XPG and restoration of the normal nucleotide sequence^11^.

In leukemogenesis, DOT1L has a well-established role in which it frequently interacts with mixed lineage leukemia (MLL) oncogenic fusion proteins, such as AF4, ENL, ELL, and AF10^12,13^, to induce H3K79 methylation and constitutively activate a leukemic transcriptional program resulting in transformation^14,15^. Inhibition of DOT1L enzymatic activity, or disruption of the interaction between DOT1L and the MLL fusion proteins, is a promising therapeutic strategy for the treatment of MLL fusion-related leukemia^16^. EPZ-5676, a small-molecule inhibitor of DOT1L, has been evaluated for MLL-rearranged leukemia in Phase I clinical trials (clinicaltrials.gov, EPZ-5676-12-001/NCT01684150/). Here tumor genome sequencing efforts have identified inactivating mutations in *DOT1L* in 4.4–15% of melanomas. Importantly, in the absence of DOT1L, UVR-induced DNA damage is inefficiently repaired such that DOT1L loss promotes melanoma development in mice after exposure to UVR.

## Results

### Identification of DOT1L mutations in human melanoma

**Fig. 1:**
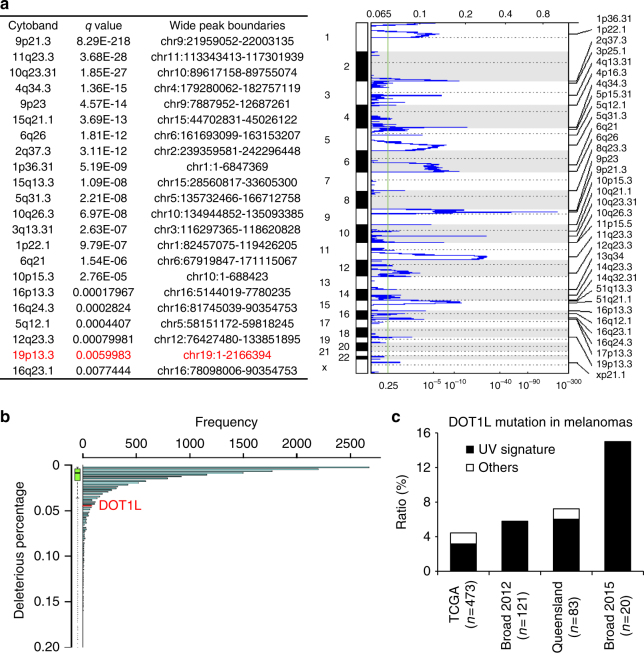
Identification of DOT1L mutations in human melanoma. **a** Somatic CNVs identified by GISTIC analysis of SNP6 data. Significantly deleted chromosome regions are shown. The deletion in red contains the *DOT1L* gene. **b** Gene-based burden test by ANNOVAR. **c** Percentages of melanoma samples with DOT1L mutations identified from the TCGA data portal, Queensland, and two published papers from the Broad Institute. DOT1L mutations from TCGA data portal and the published papers from the Broad Institute were identified by GATK and MuTect/VarScan using data from both melanoma tissues and paired germline DNA samples. DOT1L mutations from Queensland were analyzed according to the Illumina protocols

GISTIC analysis of SNP6 data for somatic copy number variation (CNV) and similar GISTIC analysis^17^ show that one common deletion region in melanoma chr19p13.3 (chr19:1-2166394) overlaps *DOT1L* (*q* value = 0.006) (Fig. 1a). Among all 83 genes in the CNV chr19:1-2166394 region, we did not find any other genes involved in the response to DNA damage based on analysis by the Gene Ontology Consortium^18^.

To explore how DOT1L might function in melanoma development, we analyzed somatic mutation data of melanomas collected from The Cancer Genome Atlas (TCGA) data portal and found 427,383 somatic variant calls made from 470 human samples (tumor samples with matched normal control) spanning 19,563 distinct genes. These included 37 somatic mutations of *DOT1L* annotated by the ANNOVAR algorithm^19^. Synonymous mutations or nonsynonymous mutations without deleterious effects as predicted by SIFT score (SIFT_Pred flag=D) were excluded^20^ leaving 23 somatic mutations with likely deleterious effects. All of them are point mutations and singletons, except one (chr19:2225413), which is detected in two samples (Supplementary Data 1). This is consistent with the finding that these mutated residues occur with extremely low variant frequency (<0.001) (Supplementary Data 2) and are usually evolutionarily conserved (GERP++ score >2).

To determine whether the rate of deleterious mutation in *DOT1L* is significantly higher than in other genes, we conducted a gene-based burden test^21^. Specifically, we created 1 supervariant for each gene by combining all the singletons and variants within it using the following rules: for each sample, (a) the deleterious flag was set to 1, if there was at least 1 deleterious somatic mutation observed; (b) otherwise, the deleterious flag was set to 0^22^. For this melanoma cohort (*n* = 470), the 19,563 genes exhibit an average deleterious mutation rate of 0.011649. For the *DOT1L* gene, we find that 21 samples have at least 1 predicted deleterious somatic mutation, namely, the deleterious rate is 21/470 = 0.045, which is significantly higher than the average (*p* value = 6.44 × 10^−8^) (Fig. 1b).

We also detected 33 missense somatic mutations using the GATK and MuTect/VarScan algorithms from both melanoma tissues and paired germline DNA samples, which is similar to the ANNOVAR algorithm (Fig. 1c and Supplementary Fig. 1). Specifically, missense DOT1L mutations were identified in 4.4% (21/473) of TCGA melanomas, 5.8% (7/121) in the Broad Institute Database (2012)^23^ and 15.0% (3/20) in the Broad Institute Database (2015)^24^ (Fig. 1c), a similar mutation rate to that observed for some well-characterized melanoma drivers, such as *IDH1* and *CDK4*^23^. Notably, 75.8% (25/33) missense mutations are C-T or CC-TT UVB signature mutations (Fig. 1c, Supplementary Fig. 1 and Supplementary Data 3).

Next, we sought to understand the biological significance of DOT1L in melanomas. We treated melanoma cells with different doses of the DOT1L inhibitor EPZ-5676 and assessed its effects on H3K79 methylation and cell growth. Consistently, we found that DOT1L inhibition reduced H3K79me2 levels (Supplementary Fig. 2a) and blocked MV411 and Molm14 MLL-rearranged leukemia cell growth in a dose-dependent manner but not the growth of human primary melanocytes (HPMs) or melanomas, including UACC62, A375, C021, C052, A04, and B16 (Supplementary Fig. 2b–d). Furthermore, we investigated the role of DOT1L absence on cell growth in non-MLL arranged leukemia HL-60 and normal HPM cells. Specifically, the *DOT1L* gene was silenced using specific shDOT1L and the resulting polyclonal cell lines were assayed for H3K79 methylation and cell growth using cell counting and 3-[4,5-dimethylthiazol-2-yl]-2,5 diphenyl tetrazolium bromide (MTT) assays. The result showed that DOT1L-targeted inhibition inhibited H3K79 methylation in all cell lines and specifically inhibited cell growth in leukemia cells with MLL translocations such as MV411, but not in melanoma cells, or leukemia without MLL translocations, such as HL-60 (Supplementary Fig. 2e). These results are consistent with previous reports that DOT1L-targeted inhibition selectively kills MLL-rearranged leukemia cells but has little impact on leukemia cells without MLL rearrangements^25^.

### DOT1L mutations in human melanoma are loss of function

**Fig. 2:**
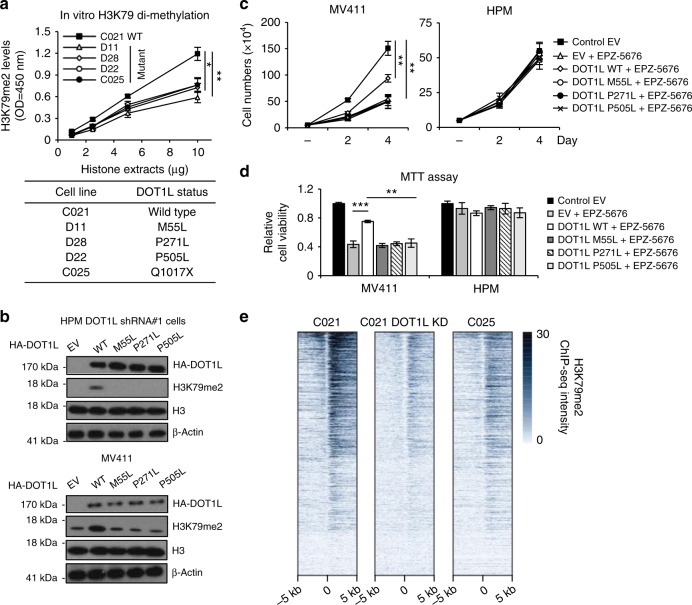
DOT1L mutants in human melanoma are loss of function. **a** In vitro H3K79me2 levels from melanoma cells with known DOT1L mutation status from Queensland, Australia. **b** DOT1L-depleted HPMs were infected with lentivirus encoding WT or mutant DOT1L as indicated. MV411 cells were transiently transfected with WT or mutant DOT1L as indicated. **c**, **d** MV411 cells or DOT1L-depleted HPMs with WT or mutant DOT1L overexpression were treated with EPZ-5676 (5 µM). Cell growth was detected using cell number counting (**c**) or MTT assay (**d**). **e** H3K79me2 signal intensities in the gene body regions, under the condition of C021 shControl, C021 shDOT1L, and C025 shControl. *p < 0.05, **p < 0.01, ***p < 0.001, unpaired Student’s *t*-test. Error bars represent ± s.d.

We then examined the effects of DOT1L mutations on H3K79 di-methylation on four cell lines, D11, D28, D22, and C025, derived from human melanomas with missense mutations. Exome sequencing of these cell lines showed that one of them, D11, has loss of heterozygosity at *DOT1L* (Supplementary Data 4). Endogenous expression of wild-type (WT) and mutant DOT1L was analyzed and comparable levels of DOT1L protein were observed in all cell lines with the exception of C025 that contains a mono-allelic truncation mutation (Supplementary Fig. 3a). Notably, levels of H3K79 methylation markedly decreased in cells with *DOT1L* mutations in comparison to cells with WT DOT1L (C021), with the DOT1L mutations detected in all the detected melanoma cell lines indicated (Fig. 2a and Supplementary Fig. 3b).

To confirm this result, we re-introduced WT or mutant DOT1L into DOT1L-silenced HPMs and then measured di-methylated H3K79. In cells that had their endogenous DOT1L silenced, expression of exogenous WT DOT1L increased H3K79 di-methylation, whereas expression of the M55L, P271L, and P505L and Q1017X mutants did not (Fig. 2b, upper panel). Similarly, expression of WT DOT1L in MV411 leukemia increased H3K79 methylation, whereas mutant DOT1L proteins failed to do so (Fig. 2b, lower panel). These results and results from similar experiments in C021, A375, and UACC62 melanoma cells as well as 293T cells suggest that DOT1L mutations found in human melanomas likely have impaired methyltransferase activity, rather than having dominant-negative effects (Supplementary Fig. 3c).

To understand the significance of the *DOT1L* mutations on their defective function, we examined the crystal structure of the DOT1L catalytic domain (1–416) solved by Min et al.^26^ showing residues 4–332 in the ordered structure. This revealed that a flexible loop and the C-terminal region of the structure is responsible for binding to the co-factor S-(5′-adenosyl)-L-methionine (SAM), which acts as the methyl group donor during methylation of the nucleosomal histones (Supplementary Fig. 3d). Based on methyl group donor (SAM) orientation and computational docking^26^, a substrate-binding interface of the C-terminal catalytic domain was proposed. Two of the mutations identified in the study, M55L and P271L, are in the *N*-terminal catalytic domain. While M55 is in the *N*-terminal region of the catalytic domain and away from the SAM binding pocket, P271 resides in the C-terminal SAM-binding region. Interestingly, M55 and P271 are also located at the interface between DOT1L and its nucleosomal histone substrate. Both mutations, M55L and P271L, retain the hydrophobicity of the positions. The M55L mutant has a longer but less bulky side chain, likely affecting the local conformation in the vicinity in a modest fashion. Mutation P271L most likely introduces extra flexibility into the backbone region. These results suggest that the modest conformational changes caused by mutations at positions 55 and 271 may affect the recognition of nucleosomal histones by the catalytic domain and consequently the catalytic efficiency of the methyltransferase. To validate this analysis, we tested whether the mutations affect DOT1L chromatin recognition and binding. Using a chromatin fractionation assay, we observed that the M55L and P271L mutations in DOT1L dramatically reduced its association with histone substrate (Supplementary Fig. 3e), suggesting that these mutations impair DOT1L in recognizing its histone substrate and thereby compromise DOT1L’s histone methyltransferase function. Similar results were obtained using the P505L mutant. While P505 is not located in the catalytic center, PolyPhen 2.2.2 analysis suggested that the P505 mutation would result in a conformational change that is likely to account for its failure to tightly associate with chromatin. These observations are consistent with our cellular studies (Fig. 2a, b).

To determine the impact of mutated DOT1L in regulating proliferation, WT and M55L, P271L, and P505L DOT1L expression constructions were introduced into MV411 leukemia cells or HPMs and cell growth after treatment with the DOT1L inhibitor EPZ-5676 was analyzed using cell counting (Fig. 2c) and an MTT assay (Fig. 2d). In MV411 cells, the expression of WT DOT1L partially reversed the effect of the inhibitor, whereas no enhanced proliferation was seen on expression of any of the mutants. By contrast, in HPMs, the DOT1L inhibitor, WT, or mutant DOT1L did not show the obvious effect on cell proliferation (Fig. 2c, d). Collectively, these results suggest that while DOT1L regulates leukemia cell proliferation it does not play a major role in regulating normal melanocyte growth.

To further explore the roles of DOT1L in melanomas, chromatin immunoprecipitation–sequencing (ChIP-seq) assays of H3K79me2 in C021 cells with and without short hairpin RNA (shRNA) silencing of DOT1L and in C025 cells bearing a DOT1L mono allelic loss-of-function mutation were performed. In agreement with previous reports^27^, the H3K79me2 ChIP-seq peaks were enriched in gene bodies, with the strongest signal observed immediately downstream of transcription starting sites (Fig. 2e). Furthermore, either silencing or mutation of DOT1L resulted in a global decrease of H3K79me2 enrichment. These results indicated that disease-associated DOT1L mutations have decreased methyltransferase activity and reduced levels of H3K79me2 across the genome.

In order to interrogate whether DOT1L has cancer-type-specific targets, we compared our results with another study that performed H3K79me2 ChIP-seq assays in a leukemia cell line MV411 with and without the treatment of the DOT1L inhibitor SGC0946^28^. In leukemia, the H3K79me2 enrichment was abnormal in amount and distribution, which is associated with rearranged MLL target loci^27,29,30^. Our study identified 1518 genes whose gene body regions contain sites with decreased H3K79me2 levels in the DOT1L-mutant melanoma cell line C025 and shDOT1L-treated cell line C021. We analyzed the data from the other study and identified 1508 genes whose gene body regions contain sites with significantly decreased H3K79me2 levels in the DOT1L inhibitor-treated leukemia cell line MV411. We refer to those genes as DOT1L-target genes. We found that 406 DOT1L-target genes (~27% for either melanoma or leukemia) are shared between the melanoma and leukemia cell lines (Supplementary Fig. 4a). For instance, DOT1L targets the *MYB* gene locus in the MV411 leukemia cells but not in the C021 or C025 melanoma cells (Supplementary Fig. 4b). This is also the case for *HOXA9* and *RUNX1*, which are well-known DOT1L-target genes in MLL-rearranged leukemia^29,31^. In contrast, DOT1L targets the DDB1 gene locus only in melanoma cell lines (Supplementary Fig. 4b). While the DDB1 gene body is enriched with H3K79me2 in both MV411 and C021 cell lines, DOT1L inhibition only results in a significant decrease in the H3K79me2 level in C021 cells. Taken together, the different pattern of DOT1L/H3K79me2 in melanoma vs. leukemia cells suggest a different role of DOT1L/methylated H3K79 in melanoma compared to leukemia.

### DOT1L mutations promote melanomagenesis with BRAF^V600E^

**Fig. 3:**
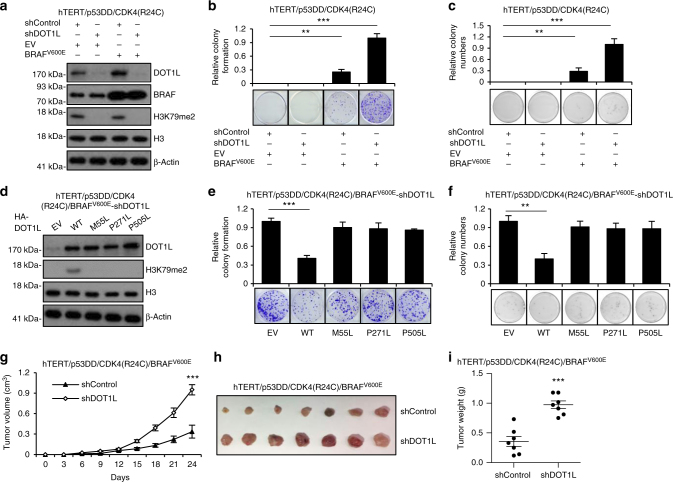
DOT1L mutations cooperate with BRAF^V600E^ to promote melanomagenesis. **a** Engineered human melanocytes were infected with the shDOT1L lentiviral construct to deplete endogenous DOT1L, with control or BRAF^V600E^ expression. **b** The cells generated in **a** were subjected to clonogenic survival assays 14 days after UVR. Crystal violet was used to stain the colonies and the relative colony formation was calculated as mean ± s.d. **c** The cells generated in **a** were seeded (10,000 cells per well) for soft agar assay and cultured for 30 days. The relative colony numbers were calculated as mean ± s.d. **d** DOT1L-depleted hTERT/p53DD/CDK4(R24C)/BRAF^V600E^ melanocytes expressing empty vector (EV), DOT1L wild-type (WT), M55L, P271L, and P505L mutants were subjected to western blot. **e** The cells generated in **d** were subjected to clonogenic survival assays 14 days after UVR. Crystal violet was used to stain the colonies and the relative colony formation was calculated as mean ± s.d. **f** The cells generated in **d** were seeded (10,000 cells per well) for soft agar assay and cultured for 30 days. The relative colony numbers were calculated as mean ± s.d. **g**–**i** Growth curves for the xenograft experiments. The indicated 3 × 10^6^ tumor cells were inoculated subcutaneously into each flank of the nude mice. The visible tumors were measured at the indicated days, error bars represent ± s.e.m, *n* = 7 (**g**). Dissected tumors (**h**) and tumor weight (**h**) were displayed. **p < 0.01, ***p < 0.001, unpaired Student’s *t*-test

To ask whether mutated DOT1L or DOT1L silencing might affect UVR-induced melanomagenesis, we used immortalized human primary melanocytes (hTERT/p53DD/CDK4(R24C))^32^ to establish polyclonal cell lines expressing shDOT1L or an shRNA control with or without the additional expression of BRAF^V600E^ (Fig. 3a). These immortalized melanocytes form anchorage-independent colonies in the presence of both BRAF^V600E^ mutation and pretreatment with low dose of UVB (20 J m^−2^)^33,34^. Those cells were subjected to clonogenic survival and anchorage-independent growth assays. Our results from the colony formation and soft agar assays revealed that BRAF^V600E^-mediated cellular transformation was dramatically enhanced by DOT1L silencing (Fig. 3b, c). We also re-introduced WT and mutant DOT1L into DOT1L-depleted hTERT/p53DD/CDK4(R24C)/BRAF^V600E^ melanocytes (Fig. 3d), with colony formation and soft agar growth assays indicating that only WT DOT1L efficiently rescued DOT1L silencing-induced cellular transformation (Fig. 3e, f). Moreover, to examine the effect of DOT1L on tumor growth in vivo, hTERT/p53DD/CDK4(R24C)/BRAF^V600E^ DOT1L-depleted melanocytes were inoculated subcutaneously into each flank of nude mice. Examination of xenograft tumor volumes and weight indicated that loss of DOT1L promoted xenograft formation (Fig. 3g–i). All results indicate that DOT1L silencing is a helper in melanomagenesis and that, in the presence of activated oncogenes, such as BRAF^V600E^, loss of DOT1L facilitates oncogenic transformation.

### UVR-induced melanoma development in DOT1L knockout mice

**Fig. 4:**
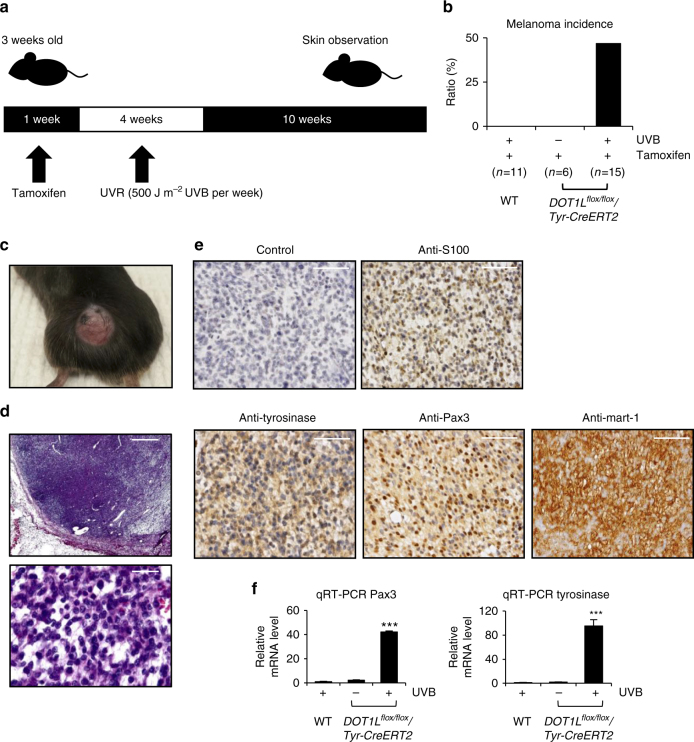
UVR-induced melanoma development in melanocyte-specific DOT1L conditional knockout mice. **a** Experimental design for UVR-induced melanoma development in the DOT1L^flox/flox^/Tyr-CreERT2 mice. **b** Melanoma incidence in wild-type and DOT1L^flox/flox^/Tyr-CreERT2 mice with or without UVB irradiation. **c** Image of a representative DOT1L^flox/flox^/Tyr-CreERT2 cutaneous melanoma. **d** H&E staining of a representative cutaneous melanoma. Scale bar, 500 μm (upper panel) and 50 μm (lower panel). **e** Immunohistochemistry staining of S100, tyrosinase, Pax3, and Mart-1 of the representative cutaneous melanoma. Scale bar, 100 μm. **f** The relative mRNA expression of Pax3 and tyrosinase in mouse melanomas comparing to those of normal skin tissues. ***p < 0.001, unpaired Student’s *t*-test. Error bars represent ± s.d.

To better define the role of DOT1L in melanocytes and UVR-induced melanoma development in vivo, we specifically depleted DOT1L expression in melanocytes using conditional *Dot1l* knockout mice (*Dot1l*^flox/flox^), in which exon 2 of *Dot1l* was floxed. Cre-mediated excision of exon 2 leads to a frame shift mutation to inactivate Dot1l^35^. Transgenic mice expressing Cre-ERT2 protein under the control of the tyrosinase (*Tyr*) promoter^36^ were crossed with *Dot1l*^flox/flox^ mice with tamoxifen administration depleting Dot1l in melanocytes leading to loss of functional Dot1l mRNA (Supplementary Fig. 5a). Mice were given a dose of 500 J m^−2^ UVB irradiation each week for 4 weeks and then incidence of melanoma was observed for another 10 weeks (Fig. 4a). WT mice (*n* = 11) were treated with both UVB irradiation and tamoxifen administration. *Dot1l*^*flox/flox*^*/Tyr-CreERT2* mice (*n* = 6) with tamoxifen administration but without UVB irradiation served as controls and no melanoma incidence was observed in the control mice. Notably, 46.7% (*n* = 7/15) of UVB-irradiated *Dot1l*^*flox/flox*^*/Tyr-CreERT2* mice with tamoxifen administration developed melanoma (Fig. 4b, c). No hyperpigmentation or nevi were found in *Dot1l*^*flox/flox*^*/Tyr-CreERT2* mice without UVB irradiation nor in the WT DOT1L mice with UVB irradiation. The melanomas closely resembled human vertical growth phase melanoma and were composed of epithelioid and spindle cells with fine pigmentation in the cytoplasm. The nuclei of the tumor cells are enlarged with some having small nucleoli, and melanophages were scattered within the tumors (Fig. 4d). Strong S-100, Melan-A/Mart-1, Tyrosinase (Tyr) and Pax3 expression was detected in all tumors (Fig. 4e). These results indicate that melanoma development in melanocyte-specific Dot1l knockout mice is UVR dependent.

To characterize the molecular characteristics of Dot1l-null melanoma, we measured the mRNA expression of neural crest development genes, tumor-suppressor genes, and oncogenes in melanoma development by quantitative reverse-transcriptase PCR (qRT-PCR). We found Pax3 and Tyrosinase mRNAs are upregulated in the mouse tumors (Fig. 4f). Together with the identification of DOT1L mutations in human melanomas, these data suggest a UVR protective role for DOT1L in melanocytes.

Our data suggest that *DOT1L* deletion alone is insufficient to induce melanoma. To identify any potential cooperation between the common oncogenic alterations with *DOT1L* mutation in melanoma development, we first analyzed the relationship between *BRaf/NRas* status and *DOT1L* status in the TCGA melanoma cohort. By using CBioPortal^37,38^ analysis (Supplementary Fig. 5b), *DOT1L* mutations were found to partially co-exist with *BRaf* and/or *NRas* mutation(s). Then we examined *BRaf* and *NRas* status in DOT1L-null mouse melanomas and found that *BRaf* (V637, homolog to human *BRaf* V600) mutations are frequently observed in melanomas from Dot1l-null transgenic mice (*n* = 4/7) (Supplementary Fig. 5c). Mutations of *NRas* (Q61, G12, and G13) were not detected in Dot1l-null mouse melanomas (Supplementary Fig. 5c).

Our data indicate that DOT1L loss of function alone is insufficient to induce melanoma development. Specifically, melanoma was observed in UVB-irradiated Dot1l-null mice, but not in unirradiated Dot1l-null mice. These data are consistent with previous reports that dysfunction of a single signaling pathway is not sufficient to induce melanoma development in vivo. For example, it is reported that 82% of benign nevi carry the same mutation in BRAF as observed in melanoma^39^. However, if another pathway is disrupted, e.g., through depletion of the tumor-suppressor gene PTEN, mice develop melanoma at age 2 months^40^. In the current study, nevi were not observed in Dot1l-null mice. Furthermore, gene expression profiling of human melanomas also indicates that, unlike BRAF mutations, DOT1L loss-of-function mutations have no correlation with the expression of MITF and PAX3, key regulators of melanoma biology.

### DOT1L is involved in NER through interacting with XPC

**Fig. 5:**
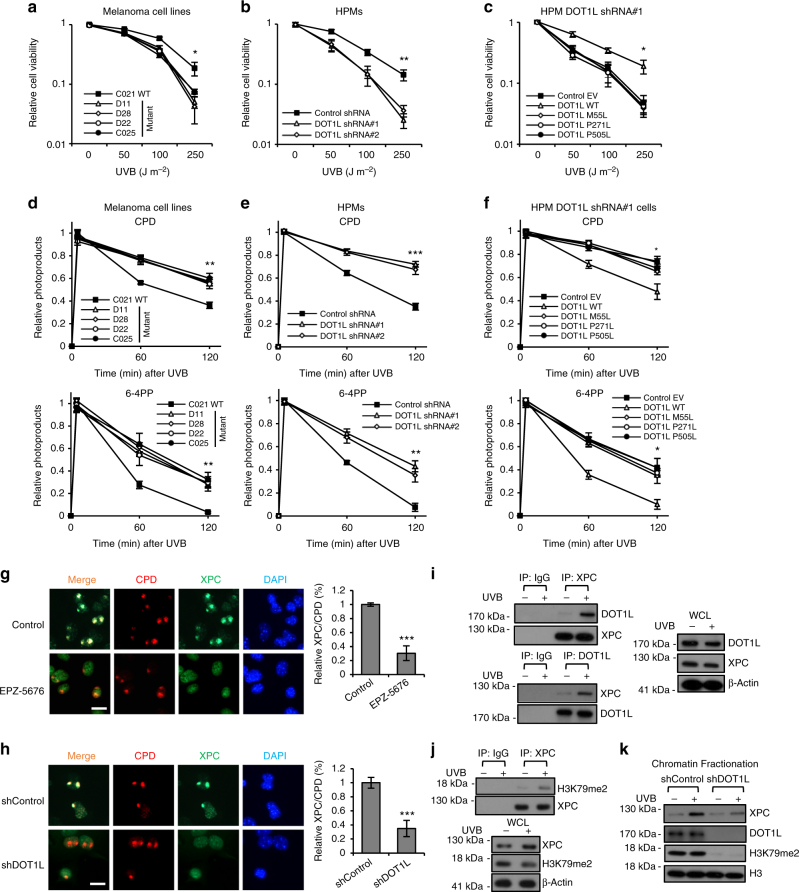
DOT1L/methylated H3K79 is involved in NER on UVB-induced DNA damage through interacting with XPC. **a** Melanomas from Queensland, Australia were irradiated with different doses of UVB. Cell viability was measured by MTT assay 24 h after UVB irradiation. **b** HPMs with stable shDOT1L or shControl were irradiated with different doses of UVB as indicated. Cell viability was measured by MTT assay 24 h after UVB irradiation. **c** WT or different DOT1L mutants as indicated was introduced into HPMs with stable shDOT1L expression. The resulting cells were irradiated with different doses of UVB as indicated. Cell viability was measured by MTT assay 24 h after UVB irradiation. **d**–**f** Melanomas from Queensland, Australia (**d**), HPMs with stable shDOT1L expression (**e**), or HPMs with stable shDOT1L expression and WT or different mutant DOT1L reintroduction (**f**) were irradiated with 100 J m^−2^ UVB and then collected at different time points as indicated after UVB irradiation. Genomic DNA was extracted and photoproducts were detected. **g**,** h** HPM cells treated with EPZ-5676 or vehicle control (**g**) or DOT1L depletion or shControl (**h**) were subjected to 100 J m^−2^ UVB under 5 µm micropore filter and were co-stained for CPD and XPC after 0.5 h. Scale bar, 20 μm. **i** The whole-cell extracts from HPM subjected to 100 J m^−2^ UVB were prepared for Co-IP assay to test the interaction of DOT1L with XPC. **j** The whole-cell extracts from HPM subjected to 100 J m^−2^ UVB were prepared for Co-IP assay to test the interaction of XPC with H3K79me2. **k** HPMs with DOT1L depletion or shControl were exposed to 100 J m^−2^ UVB. After 0.5 h, the chromatin fraction was prepared for western blot. *p < 0.05, **p < 0.01, ***p < 0.001, unpaired Student’s *t*-test. Error bars represent ± s.d.

Next, we focused on the role of DOT1L in the UVR response in melanocytes, as almost all DOT1L mutations are UV signature mutations (Fig. 1c and Supplementary Data 3), and UV irradiation is required in Dot1l-null-driven melanoma development (Fig. 4b). To identify how DOT1L protects against UVR in melanocytes, we examined UV response of melanoma cells with WT or different DOT1L mutants and found that melanoma cells with mutant DOT1L are more sensitive to UV irradiation than cells with WT DOT1L (Fig. 5a). Next, HPMs with DOT1L silencing were irradiated with different doses of UVB and cell viability was determined using the MTT assay 12 h after UVR. Similarly, HPMs silenced for DOT1L were also more sensitive to UVB irradiation (Fig. 5b). Moreover, re-expression of WT but not mutant DOT1L in DOT1L-silenced HPMs increased viability following UVR compared to re-expression of DOT1L mutants (Fig. 5c). Consistent with the protective role of DOT1L in UVR response, treatment of melanoma cells with the DOT1L inhibitor EPZ-5676 decreased cell viability following UVR (Supplementary Fig. 6a). Collectively these data suggest that DOT1L is a protective factor for UVR in melanocytes.

The increased sensitivity of DOT1L mutant cell lines to UVR suggested that DOT1L-mediated H3K79 methylation may have a role in DNA repair following UVR-induced damage. UVB irradiation mainly induces two types of DNA lesions (damage), 6–4 PPs and CPDs^41^. Using enzyme-linked immunosorbent assays, we found that CPDs and 6–4 PPs photoproducts were significantly less efficiently repaired in melanoma cells with DOT1L mutations than that in those with WT DOT1L (Fig. 5d and Supplementary Fig. 6b). To determine whether there is a direct involvement of DOT1L/methylated-H3K79 in DNA repair after UVR exposure, we also assessed whether DOT1L silencing or inhibition influences DNA repair after UVB irradiation in HPMs. CPDs and 6–4 PPs were measured after UVB irradiation (100 J m^−2^) in isolated HPMs stably expressing shDOT1L. Stable expression of two different shRNAs directed against DOT1L in HPMs led to a significantly reduced capacity to repair UV-induced DNA damage (Fig. 5e) compared to cells expressing a control shRNA. To eliminate the possibility of off-target effects associated with shRNA treatment, we reintroduced WT-DOT1L or mutant DOT1L (M55L, P271L, or P505L) into DOT1L-depleted cells and found that WT but not mutant DOT1L restored efficient DNA repair (Fig. 5f). Similar results were obtained using the DOT1L inhibitor EPZ-5676 (Supplementary Fig. 6c). CRISPR/Cas9 engineered DOT1L knockdown in HPMs and mouse B16 melanoma cells, which were pooled as heterogeneous transfected cells after drug selection, also failed to affect cell viability (Supplementary Fig. 7a, b) unless cells were UVB irradiated (Supplementary Fig. 7c). Sensitivity of the CRISPRR/Cas9-mediated DOT1L knockdown cells correlated with decreased capacity to repair CPDs and 6–4 PPs (Supplementary Fig. 7d, e). Together, these results indicate that DOT1L facilitates DNA repair after UVB irradiation in vitro.

Studies show that Dot1 and H3K79 methylation are required for NER with yeast, in which methylated H3K79 seems to serve as a docking site for the repair machinery on the chromatin^42^. In addition, NER Dot1l-dependent H3k79me has been indicated to potentially function in yeast to coordinate various repair processes, such as heterochromatin-mediated silencing, post-replication^43^. However, in mouse embryonic fibroblasts Dot1l is required at the transcription recovery stage following the removal of damaged DNA, but not during the repair process^44^. The specific function of DOT1L and methylated H3K79 in NER in mammalian cells such as melanocytes therefore remains unclear.

To decipher the role of DOT1L in melanocyte DNA repair, we first asked whether its expression was induced by UVB irradiation. Specifically, HPMs were irradiated with 100 J m^−2^ UVB and the expression of DOT1L and methylated H3K79 were detected. UVR-induced p53 upregulation served as a positive control. Interestingly, the expression of DOT1L protein and methylated H3K79 were stable at different time points after UVR while p53 expression was robustly upregulated (Supplementary Fig. 8a). These results indicate that DOT1L/methylated H3K79-regulated DNA repair is not mediated through the upregulation of DOT1L expression.

NER is an important DNA repair mechanism that removes UVR-induced DNA damage^45^. Next, we measured the levels of some NER factors (including XPA, XPC, DDB1, DDB2, ERCC1, and p62) after UVB irradiation in melanocytes with stable DOT1L silencing to determine whether DOT1L/methylated H3K79 regulates the expression of co-factors in DNA repair (Supplementary Fig. 8b). We found that UVB irradiation did not alter the expression of NER factors. We also found that the protein expression of XPA, XPC, DDB1, DDB2, ERCC1, and p62 were DOT1L and methylated H3K79 independent (Supplementary Fig. 8b). These results suggest that DOT1L-regulated DNA repair is not mediated through the transcription regulation of these genes implicated in NER^9^, a conclusion substantiated using gene array analysis (see below).

As DOT1L does not appear to regulate the expression of DNA repair factors, we therefore assessed whether DOT1L is required for the recruitment of NER proteins to sites of damage. We stably knocked down DOT1L or used a DOT1L inhibitor EPZ-5676 in HPMs, and then irradiated those cells with 100 J m^−2^ UVB under a 5 µm micropore filter^46,47^. Then we co-stained the cells with antibodies against CPD and NER proteins including DDB1, DDB2, XPC, XPA, ERCC1, and p62. Our results showed that the co-localization of DDB1/DDB2 with CPD was not affected by DOT1L knockdown, while the recruitment of XPC, XPA, ERCC1, and p62 to CPD were strongly suppressed (Fig. 5g, h and Supplementary Fig. 8c). These data suggested that DOT1L/H3K79 methylation is required for recruitment of XPC and its downstream factors XPA and p62 in NER in response to UV irradiation. In addition, to confirm the role of DOT1L on recruiting XPC, we immunoprecipitated DOT1L in HPMs treated with100 J m^−2^ UVB exposure, and the interaction between DOT1L and XPC was detected (Fig. 5i). To identify whether DOT1L-depdendent H3K79me2 also forms complex with XPC, we also performed co-immunoprecipitation in UV-radiated HPMs. We found that XPC interaction with H3K79me2 is in a UVB-dependent manner (Fig. 5j). We also performed the chromatin fractionation assays to determine whether the binding of XPC to damaged DNA chromatin is dependent on DOT1L. The results showed that DOT1L was required for efficient XPC recruitment to chromatin in response to UVB-induced DNA damage (Fig. 5k). Taken together, these results showed that UVB exposure led to the formation of the complex that consisted of DOT1L/methylated H3K79 and XPC. In addition, we found that DOT1L recruitment on chromatin was not changed in response to UVB-induced DNA damage (Fig. 5k). This result is consistent with the data that methylation of H3K79 was not changed in response to DNA damage (Supplementary Fig. 8a–b). We further showed that the interaction with XPC was mediated by DOT1L amino acids 580–1138 (Supplementary Fig. 8d). These data suggest that DOT1L interacts with XPC and is required for DNA damage repair. Taken together, these results suggest that DOT1L and H3K79 methylation provided a template for XPC to perform NER in melanocytes.

A recent report demonstrated a direct interaction between histone and XPC, through which histone deacetylation modulates the localization and functions of XPC in the process of DNA damage recognition for NER^48^. Here we show that DOT1L/H3K79 methylation also plays the crucial role on the regulation of XPC recognition of DNA lesion and modulates the interaction between XPC and histones. Previous work indicates that methylation of H3K79 potentially modulates the nucleosome surface, leading to changing interactions of macromolecules with the nucleosome^49^, which is consistent with our discovery that DOT1L-dependent H3K79 methylation modulates XPC interaction with histones and recognition of UVR-induced DNA damage site for NER. Together these data indicate that histone modification-related chromatin structure is essential for XPC function, and the histone modification is crucial in the process of DNA damage recognition for NER. However, the specific underlying molecular mechanisms still require further investigation.

To further clarify the connection between DOT1L/methylated H3K79 and DNA repair, we analyzed our ChIP-seq data to identify the DNA regions that show different levels of H3K79me2 between C021-shControl and C021-shDOT1L as well as between C021-shControl and C025-shControl. Specifically, enrichment analysis for the H3K79me2 signal was performed by MACS2 and significant difference was determined by log-transformed likelihood ratio (logLR) (logLR cutoff is 3, i.e. likelihood ratio = 1000)^50^. This yielded 4425 regions with significantly reduced H3K79me2 levels in C021-shDOT1L and 1947 regions with significantly reduced H3K79me2 levels in C025-shControl, of which 1647 regions have reduced H3K79me2 level in both conditions. Thus the majority of downregulated H3K27me2 sites found in C025-shControl (DOT1L mutant) are also observed with decreased H3K79me2 levels in C021 cells treated with shDOT1L. Next, we identified 1518 genes whose gene body regions contain any of the 1647 regions with reduced H3K79me2 levels in both DOT1L-mutant C025 and shDOT1L-treated C021 cells (some of the genes are associated with multiple enriched regions). Gene Set Enrichment Analysis^51^ that extracted the gene sets from the KEGG database^52^ revealed that genes were significantly enriched in multiple pathways (Supplementary Fig. 9), and the number of genes observed in each gene set and the associated *p* value is listed (Supplementary Data 5). As DNA repair must take place in the context of chromatin, these H3K79me2 enriched genes may suffer from DNA damage if DOT1L is inhibited or mutated. DNA damage in these important pathways may contribute to oncogenesis. Examples of enrichment of H3K79me2 at a selection of individual genes such as *DDB1* and *RAD23A* are presented in Supplementary Fig. 10.

In addition, we profiled gene expression by microarray for C021 with DOT1L knockdown, C025 with shControl, and EPZ-5676-treated C021 with shControl. The results showed that C021 had a different transcriptional profile of NER genes in comparison to that of C025, which might be caused by cell line specificity as they are derived from different melanoma patients. However, in the same cell line background, the DOT1L knockdown had only a very modest impact on NER gene expression (fold change from −1.3 to 1.2), a result supported by the gene expression profile from C021 cells treated with the DOT1L inhibitor EPZ-5676 (Supplementary Data 6). Specifically, we obtained log_2_-transformed expression values for 23786 Entrez Genes using Human Gene 2.0 ST arrays as described in the Methods. We found that 10,117 genes (42.5%) were significantly differentially expressed (false discovery rate (FDR) *q* < 0.05) between the C025 and C021 cells, which were derived from different melanoma patients. We also performed another microarray assay with C021-shDOT1L and C021-shControl and found 1656 genes (6.96%) with significantly differential expression (FDR *q* < 0.05). Among those, 1010 genes (4.25%) were downregulated more than two folds by DOT1L knockdown, which include *CYP21A2*,* BANCR*,* CST1*,* MPZ*, and *OAS1* with genes implicated in metabolic pathways representing the largest set of DOT1L-targeted genes. Using KEGG Pathway(s) analysis, we found that 129 genes in metabolic pathways were repressed for more than two fold, including *CYP21A2* (−2.4 fold), *QPRT* (−2.3 fold), *ALDH1A1* (−2.2 fold), and *TM7SF2* (−2.0 folds). Our microarray also found that 19 genes associated with cell adhesion molecules are potentially controlled by DOT1L at the transcriptional level and 6 genes in cell cycle (Supplementary Data 7). However, no critical genes implicated in NER were found to be regulated by DOT1L. The microarray data can be accessed in the Gene Expression Omnibus (GEO) in Series GSE103079 and GSE103080. These data support our conclusion that DOT1L is involved in DNA repair through recruiting the NER factors onto DNA damage sites but is unlikely to affect repair through regulation of NER genes at the transcriptional level.

## Discussion

Dot1l and H3K79 methylation may serve as a docking site for the repair machinery on chromatin and thus is required for yeast NER^42^. Further research has confirmed this function in NER and also found that Dot1l and H3K79 methylation potentially play multiple roles in the response to UV damage in yeast, including post-replication and checkpoint function^43^. On the other hand, in mouse embryonic fibroblasts Dot1l is required at the transcription recovery stage following the removal of damaged DNA but not the repair process^44^. Here our data indicate that, in melanocytes and melanoma, DOT1L and H3K79 methylation is required for XPC recruitment and efficient NER, and loss of DOT1L leads to UV sensitivity and inefficient removal of UV photoproducts. DOT1L therefore plays an essential role in UVR-stressed melanocytes to protect against the transition to melanoma. In addition, our work on melanocytes with conditional knockout of Dot1l provides an UVR-dependent mouse model of melanoma.

Our results suggest that DOT1L is similar to another histone methyltransferase, Enhancer of Zeste Homolog (EZH2), in cancer^53^. As a *polycomb* protein, the role of EZH2 depends on cofactors that differ by cell context and dosage^53^. In addition, the oncogenic activity of EZH2 in castration-resistant prostate cancer cells is *polycomb* independent but dependent on EZH2 phosphorylation at Ser^54^. Although DOT1L is not a typical *polycomb* protein, its role is dependent on its binding partners and cellular context. While inhibition of EZH2 reduced reprogramming efficiency and repressed the yield of induced pluripotent stem cell (iPSC) colonies, inhibition of DOT1L by shRNA or a small-molecule-accelerated reprogramming significantly increased the yield of iPSC colonies^55^. Whereas DOT1L interacts with MLL oncogenic fusion proteins, such as AF4, ENL, ELL, and AF10^12,13^ to activate a proliferative transcriptional program in leukemia cells, in melanocytes, DOT1L interacts with XPC to facilitate DNA repair and prevent melanoma development.

To study melanoma biology, a number of suspected genetic abnormalities involved in human melanoma susceptibility and tumor progression have been exploited in mice. In comparison to the Tyr-SV40E^56^ and Hgf/Sf mouse models^57^, the Dot1l conditional-null mouse model is much more efficient for the development of melanoma, with melanoma being diagnosed in nearly 50% of mice by 10 weeks after UVB irradiation. Furthermore, melanomas in UVB-irradiated Dot1l-null mice closely mimic the vertical phase of human melanoma histologically. The Dot1l-null mouse strain is therefore complementary to the BRaf^V600E^ knock-in mouse melanoma model^58^ for melanoma studies and will allow a better understanding of melanoma etiology, especially sun-related melanoma studies (both pathologic and physiologic) in ways that cannot be readily addressed with cell culture-based studies alone.

## Methods

### Cell lines, animals, and UV exposure

All cell lines were from ATCC, authenticated by ATCC, and mycoplasma negative. Cell lines and UV exposure were as described^59^. Briefly, Cells were washed by phosphate-buffered saline (PBS) twice before UVB irradiation. For the in vitro UV experiments, cells were exposed to UVR in the Stratalinker UV chamber (Stratagene, Cedar Creek, TX, USA) with UVB bulbs (UVP LLC, Upland, CA, USA). UV emittance was measured with the use of a UV photometer (UVP LLC, Upland, CA, USA)^59^. Adherent cells were irradiated through a small volume of PBS at a dose of 100 J m^−2^. An UVB dose of 100 J m^−2^ is equivalent to one standard erythema dose of UVB (SED), commonly used as a measurement of sunlight. As a reference, the ambient exposure over an entire sunny summer day in Europe (Switzerland) is approximately 30–40 SED^60^. Human primary melanocytes were isolated from normal discarded foreskins as described^61^ and were cultured in Medium 254 (Thermo Fisher Scientific Inc., Waltham, MA, USA). DOT1L^flox/flox^ mice were provided by Dr. Jay Hess^35^. Briefly, the DOT1L^flox/flox^ construct was designed such that three loxP sites were introduced into DOT1L exons containing DOT1L catalytic domain^8,62^. Transgenic mice expressing Cre-ERT2 protein under the control of the tyrosinase (Tyr) promoter were purchased from Jackson Laboratory^36^. Mice underwent tamoxifen treatment to silence DOT1L expression in melanocytes. Briefly, mice at 3 weeks of age were administered with tamoxifen in corn oil daily by intraperitoneal injection (Sigma, CAT#T5648) at 0.12 mg g^−1^ body weight for 5 consecutive days. The control mice will get plain corn oil (vehicle of tamoxifen) injection. Mouse UV exposure procedure were performed as described^33^. Briefly, a UVB dose of 500 J m^−2^ was used. This does is equivalent to one minimum erythema dose of UVB and 5 SED, commonly used as a measurement of sunlight in vivo.

None of the experiments exceeded the limit, of which maximal tumor size is 20 mm at the largest diameter permitted by IACUC of Boston University Medical Campus. Mice were maintained in a specific pathogen-free facility of the Animal Science Center of Boston University Medical Campus, and the experiments were performed according to the Institutional Animal Care and Use Committee of Boston University Medical Campus. Mice were housed on a time cycle of 12 h of light (beginning at 0600 hours) and 12 h of dark (beginning at 1800 hours). Mice were allowed free access to an irradiated diet and sterilized water. The mice were monitored daily for signs of health status and distress. Mice aged from 8 weeks to 6 months were used for breeding and pregnant females were placed in a new cage. Mice were weaned at 20–21 days of age. DNA was extracted from the mouse tail biopsy for genotyping. NCr nude mice (female, 8–10 weeks) were purchased from Taconic Biosciences.

### Real-time qPCR

The cDNA (40 ng) was used for qPCR amplification with SYBR green PCR master mix (Applied Biosystems, Invitrogen, Thermo Fisher Scientific Inc.). Samples in which no reverse transcriptase was added were included for each RNA sample. The relative levels of expression of genes were normalized according to those of *GAPDH*. Real-time qRT-PCR data were calculated using the comparative Ct method as previously described^63^. All qPCR was performed in triplicate, and three independent RNA samples were assayed for each time point. The primers for determining relative Pax3, Tyrosinase, hDOT1L, and mDot1l transcript levels were: mPax3 forward: 5′-TTTCACCTCAGGTAATGGGACT-3′, reverse: 5′-GAACGTCCAAGGCTTACTTTGT-3′; mTyrosinase forward: 5′-CGATGGAACACCTGAGGGAC-3′, reverse: 5′-TGTTCAAAAATACTTCCAGTGTGT-3′; hDOT1L forward: 5′-GAGACCTCCTTCGACCTGGT-3′, reverse: 5′-CGACGCCATAGTGATGTTTGC-3′; and mDot1l forward: 5′-CAACTGCAAACATCACTACGGA-3′, reverse: 5′-TCACCTCGTTCCAGTGTGTAT-3′.

### Bioinformatics analysis

SNP6 data were analyzed by GISTIC2.0. DOT1L mutations from TCGA were annotated by ANNOVAR, GATK, and MuTect/VarScan. Mutation damage effects were predicted using SIFT 4.0.5 or PolyPhen 2.2.2. DOT1L mutations from Queensland, Australia were analyzed using two NGS platforms (Illumina Genome Analyzer II and Life Technologies SOLiD) and mapped reads with platform-appropriate alignment programs^64^.

### Microarray

All arrays were performed by BU Microarray Sequencing Resource. Specifically, C025 cells (DOT1L mutation) and C021 cells (DOT1L WT) treated with DOT1L inhibitor, EPZ-5676, or vehicle control were exposed to 100 J m^−2^ UVB, and 3 h later the RNA was prepared with the RNeasy Mini Kit (74104) (Qiagen, Hilden, Germany) for microarray assay. C021 cells with DOT1L knockdown and Scramble control were used for RNA extraction with the RNeasy Mini Kit (Qiagen, Hilden, Germany) for microarray assay. The arrays were normalized together using the Robust Multiarray Average algorithm and a CDF (Chip Definition File) that maps the probes on the array to unique Entrez Gene identifiers. The expression values are log2-transformed by default. The technical quality of the arrays was assessed by two quality metrics: Relative Log Expression (RLE) and Normalized Unscaled Standard Error (NUSE). For each sample, median RLE values >0.1 or NUSE values >1.05 are considered out of the usual limits, although RLE is the quality metric most strongly associated with technical quality. All arrays had median RLE and NUSE values well within these limits. Principal Component Analysis was then performed. Benjamini–Hochberg FDR correction was applied to obtain FDR-corrected *p* values (*q* values), which represent the probability that a given result is a false positive based on the distribution of all *p* values on the array. In addition, the FDR *q* value was also recomputed after removing genes that were not expressed above the array-wise median value of at least one array.

### ChIP-seq analysis

ChIP-seq was performed as previously described^65^. Briefly, cells were first cross-linked by 1% formaldehyde and then lysed. The chromatin extract was sonicated for 15 min using a Diagenode bioruptor and then pulled down by an antibody recognizing H3K79me2 (Abcam ab3594) with 2 µg for each IP. Enriched DNA was released by de-crosslinking at 65 degree for 6 h and extracted by the Qiagen MinElute PCR Purification Kit (Qiagen 28006). DNA sequencing libraries were generated using the NEB ChIP-seq Library Preparation Kit (E6200S) and sequenced on an Illumina MiSeq (50-bp single-end reads). Sequencing reads were aligned to the hg19 reference genome by the Burrows–Wheeler Aligner^66^, and H3K79me2-binding sites were called by MACS2^50^. Differential binding peaks were called by MACS2 “bdgdiff” function and their closest genes were called by the GREAT pipeline^67^. Two biological replicates were included for each condition. The ChIP-seq data have been deposited at GEO (GSE89029).

### Plasmids and RNA interference

HA-DOT1L were generously provide by Dr. Yi Zhang, HHMI and Harvard^12^. The mutant DOT1L constructs were generated using the QuikChange II Site-Directed Mutagenesis Kit (Agilent Technologies, Santa Clara, CA). LentiCRISPR v2 constructs for human DOT1L or mouse Dot1l were generated following the online guide provided by Dr. Feng Zhang, Broad Institute and MIT (http://crispr.genome-engineering.org/)^68^. LentiCRISPR v2 constructs specific for human DOT1L or mouse Dot1l were transfected into HPM or B16 cells by lentivirus infection. The cells were selected with puromycin and then pooled as heterogeneous transfected cells. Ambion Silencer Select DOT1L (4392420) siRNA were purchased form Thermo Fisher Scientific Inc. (Waltham, MA, USA). The HA-DOT1L truncations were generated with the following primers (5′ to 3′): (1–586) fraction, forward, ATAGTAGAATTCACATGGGGGAGAAGCTGGAGC, reverse, ATAGTACTCGAGCTACTAGTCCTGCTCCAGCTGCTCCGACTGC; (580–1138) fraction, forward, ATAGTAGAATTCACCAGTCGGAGCAGCTGGAGCAGGAC, reverse, ATAGTACTCGAGCTACTACAGGGGCTGGTTGATGTTACTGACCATC; (1131–1537) fraction, forward, ATAGTAGAATTCACGTCAGTAACATCAACCAGCCCCTG; reverse, ATAGTACTCGAGCTACTAGTTACCTCCAACTGTGCCGCCTGCCAC; (1–1138) fraction, forward, ATAGTAGAATTCACATGGGGGAGAAGCTGGAGC, reverse, ATAGTACTCGAGCTACTACAGGGGCTGGTTGATGTTACTGACCATC; and (580–1537) fraction, forward, ATAGTAGAATTCACCAGTCGGAGCAGCTGGAGCAGGAC, reverse, ATAGTACTCGAGCTACTAGTTACCTCCAACTGTGCCGCCTGCCAC. shRNA constructs targeting human DOT1L (RHS4533-EG84444) were purchased from OpenBiosystems. The most efficient knockdown cell lines with shDOT1L (target sequence: 5′-ATAGCGAGCTTGAGATCCGGG-3′) were used in assays.

### Transfection, lentiviral, and retroviral infections

To generate stable knockdown of DOT1L in B16 and HPMs, mouse- or human-specific shRNAs in PLKO1 against DOT1L were co-transfected with psPAX2 (Addgene #12260) and pMD2.G (Addgene, #12259) in HEK293-FT (ATCC) using Lipofectamine 2000 (Invitrogen, Thermo Fisher Scientific Inc). Lentiviruses were collected after 48 h and then infected cells for 24 h in the presence of polybrene (8 μg mL^−1^) and selected with puromycin (2 μg mL^−1^). To generate cells with stable expression of WT DOT1L and mutant DOT1L, HEK293T cells were co-transfected with DOT1L constructs in pQCXIP, VSV-G, and pUMVC (Addgene #8449) plasmids using Lipofectamine 2000. Retroviruses were harvested after 48 h and cells with shDOT1L were infected with retroviruses in the presence of polybrene (8 μg mL^−1^). After 24 h, cells were selected with puromycin (2 μg mL^−1^).

### Clonogenic survival and soft agar assays

The clonogenic survival and soft agar assays for hTERT/p53DD/CDK4(R24C) melanocytes were performed as described^32^. Briefly, hTERT/p53DD/CDK4(R24C) melanocytes with or without DOT1L depletion were treated with 20 J m^−2^ UVB for 14 days before plating into six-well plate at 1000 cells per well. For soft agar assays, cells (10,000 per well) were seeded in 0.5% low-melting-point agarose in Dulbecco’s modified Eagle’s medium (DMEM) with 10% fetal bovine serum (FBS) and layered onto 0.8% agarose in DMEM with 10% FBS. The plates were kept in the cell culture incubator for 30 days after which the colonies >50 μm were counted under a light microscope. All results were calculated by three independent experiments.

### Immunoblot analysis

The following antibodies used in Western blotting were purchased from Cell Signaling Technology (Danvers, MA, USA): anti-HA-tag (6E2) (1:1000, #2367, Cell Signaling Technology), anti-p53 (7F5) (1:1000, #2527, Cell Signaling Technology), anti-DDB-1 (D4C8) (1:1000, #6998, Cell Signaling Technology), anti-DDB-2 (D4C4) (1:1000, #5416, Cell Signaling Technology) anti-ERCC1 (1:1000, #3885, Cell Signaling Technology), and anti-XPC (D1M5Y) (1:1000, #14768, Cell Signaling Technology). The Polyclonal anti-KMT4/DOT1L (1:2000, ab72454, Abcam) antibody, anti-XPA (1:2000, ab85914, Abcam), polyclonal di-methylated H3K79 antibody (1:1000, ab3594, Abcam), polyclonal to mono-methylated H3K79 antibody (1:500, ab2886, Abcam), and polyclonal tri-methylated H3K79 antibody (1:1000, ab2621, Abcam) were purchased from Abcam. TFIIH p62 Antibody (1:500, Q-19) was purchased from Santa Cruz Biotechnologies, Inc. Other antibodies include monoclonal Anti-β-Actin-Peroxidase antibody (1:25000, AC-15, Sigma Aldrich), monoclonal ANTI-FLAG® M2 antibody (1:1000, Sigma Aldrich), and anti-H3 (1:500, 865R2, Thermo Fisher Scientific Inc.). Most important uncropped blot images are shown in Supplementary Fig. 11.

### Immunohistochemistry

Tissue samples for immunohistochemistry analysis were sectioned at 4 µm thick and underwent antigen heat retrieval using EDTA 105 °C for 10 min. Antibodies include: anti-S-100 (1:100, Dako North America, Inc. Carpinteria, CA, USA), anti-Tyrosinase (1:100, T311, Dako North America), anti-Pax3 (1:100, 38-1801, Thermo Fisher Scientific Inc.), and Melan-A/MART-1 antibody (1:500, M2-9E3, Novus Biologicals, USA). Negative controls were processed in parallel using an identical protocol but with the omission of the primary antibody. Dako EnVision+ System HRP Kit was used in immunohistochemistry.

### Immunofluorescence

Immunofluorescence were performed according to standard protocols with the following antibodies: anti-XPC (1:200, D1M5Y, Cell Signaling), anti-XPA (1:200, ab85914, Abcam), anti-CPD (1:50, KTM53, Kamiya Biomedical Company), anti-TFIIH p62 (1:50, Q-19, Santa Cruz Biotech) anti-DDB-1 (1:100, ab97522, Abcam), anti-DDB-2 (10 µg mL^−1^, ab51017, Abcam), and anti-ERCC1 (1 µg mL-1, ab2356, Abcam). All slides were cover-slipped using Vectashield-DAPI mounting media (Vector laboratories, Burlingame, CA, USA). Images per sample were captured using the Leica SP5 Confocal Microscope at BU Cellular Imaging Core. To determine the relative fluorescence at local irradiated sites, the CPD-positive area was gated by the Image J software and fluorescence intensity was quantified. Then the fluorescence intensity of XPC at the same area was quantified and divided by the CPD fluorescence. Relative fluorescence was calculated as the experimental fluorescence divided by the control fluorescence^69^.

### Co-immunoprecipitation and chromatin fractionation assay

Briefly, cells were washed twice with ice-cold PBS and lysed in lysis buffer containing 50 mM Tris pH 7.4; 1% Triton X-100; 0.5 mM EDTA; 0.5 mM EGTA; 150 mM NaCl; 10% Glycerol; 1 mM phenylmethylsulfonyl fluoride, and complete protease inhibitor cocktail (Roche) on ice for 30 min. The supernatant was collected after centrifugation at 15,000 × *g* for 15 min at 4 °C, and 500 µg of total cell lysate was treated by DNase (15 U mL^−1^, Pierce), precleared by 20 µL Protein G Agarose Beads (Thermo Fisher Scientific Inc.), and then incubated with primary antibody, including anti-DOT1L (ab72454, Abcam) and anti-XPC (#14768, Cell signaling) overnight at 4 °C. In all, 20 µl of Protein A/G Agarose Beads was added into the samples with rotation at 4 °C for 1 h. After three washes with 1 mL of lysis buffer, the bound proteins were released by boiling in 30 μL of sodium dodecyl sulfate loading buffer and detected as described above. The chromatin fractionation was extracted with the Chromatin Extraction Kit (ab117152, Abcam) from the cells and assayed with western blot.

### **In vivo** tumorigenesis assay

In vivo tumorigenesis assay of hTERT/p53DD/CDK4(R24C)/BRAF^V600E^ melanocytes was performed as described previously^32^, Briefly, 3 × 10^6^ control or DOT1L-depleted hTERT/p53DD/CDK4(R24C)/BRAF^V600E^ melanocytes were mixed with matrigel (1:1) and injected subcutaneously into the flanks of female nude mice. Tumor size was measured every 3 days with a caliper, and the tumor volume was determined as mentioned above. The tumors were dissected to measure their weights 24 days after cell inoculation.

### Enzyme immunoassay

Enzyme immunoassay was performed using the anti-CPD (MC-062) and anti-6–4 PP (KTM-50) antibodies. Briefly, the heat-denatured genomic DNA was coated onto the microplate wells with Pierce DNA Coating Solution (17250, Thermo Fisher Scientific Inc.). After washing and blocking by PBS with the purified anti-mouse IgG mAb of 2 µg mL^−1^, the specific antibodies of anti-CPD (MC-062) and anti-6–4 PPs (KTM-50) were diluted 1:1000 in blocking buffer and added to each well. Optical density at 405 nm was determined.

### Histone extraction and in vitro H3K79me2 assay

Cells (1 × 10^7^) were used for the total histone extracts. EpiQuik Total Histone Extraction Kit (OP-0006) and EpiQuik Global Di-Methyl Histone H3K79 Quantification Kit (P-3056) were purchased from Epigentek Group Inc. (Farmingdale, NY, USA). Experiments were carried out following the protocols provided by the company.

### Statistical analysis

All quantitative data were presented as the mean ± SEM or the mean ± SD as indicated of at least three independent experiments by Student’s *t*-test for between-group differences.

### Data availability

All data that support the findings of this study are available on reasonable request from the corresponding author. The contributing authors declare that all relevant data are included in the paper and its supplementary information files. The microarray data can be accessed in the GEO in Series GSE103079 and GSE103080. The ChIP-seq data have been deposited at GEO (GSE89029).

## Electronic supplementary material

Supplementary Information

Description of Additional Supplementary Files

Supplementary Data 1

Supplementary Data 2

Supplementary Data 3

Supplementary Data 4

Supplementary Data 5

Supplementary Data 6

Supplementary Data 7
