# Supplementary material for: The protective role of DOT1L in UV-induced melanomagenesis
Source: Nat Commun. 2018 Jan 17;9:259. doi: 10.1038/s41467-017-02687-7 (PMC5772495; doi:10.1038/s41467-017-02687-7)
Supplement: Supplementary file 1 — Supplementary Information [file 41467_2017_2687_MOESM1_ESM.pdf]

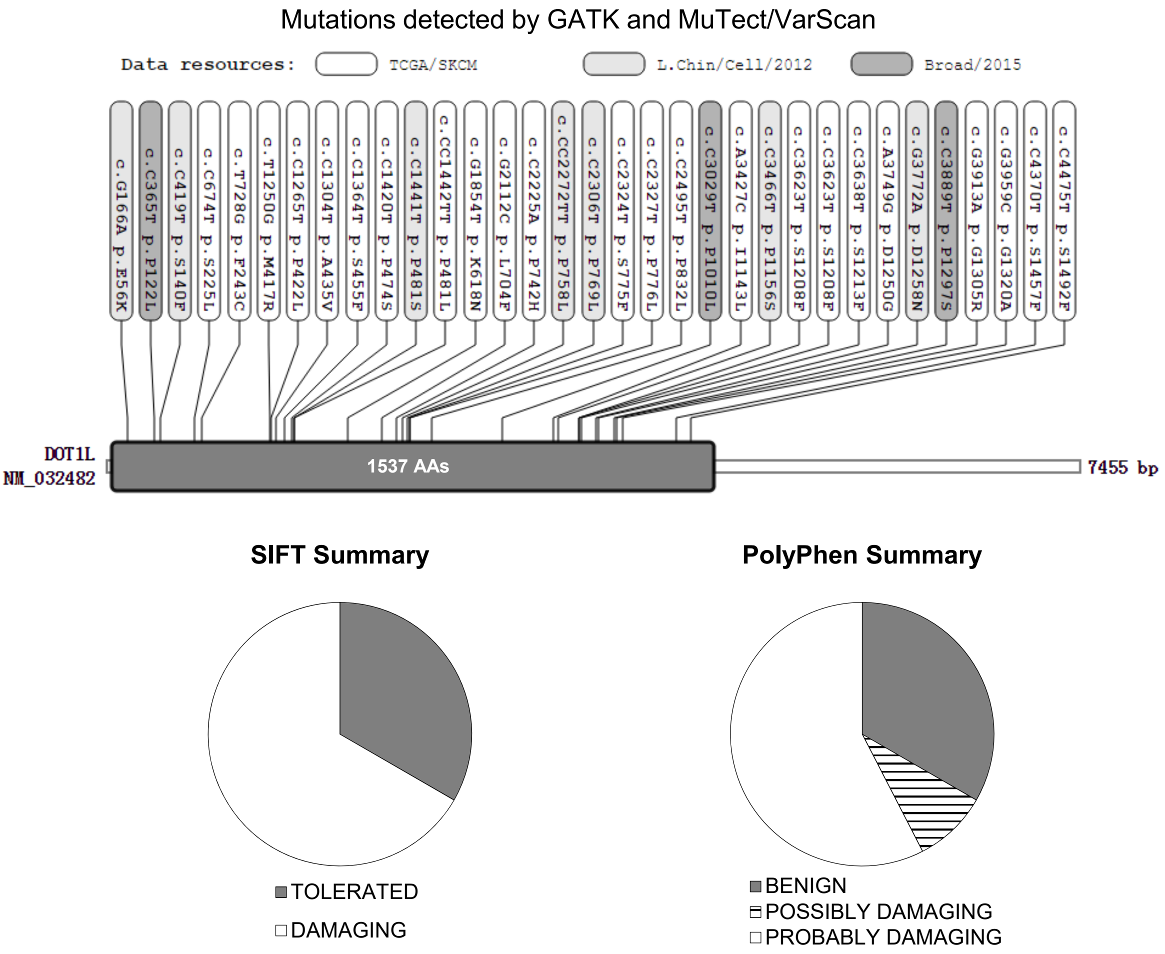

Supplementary Figure 1. Identification of DOT1L mutations in human melanomas.
Graphic display of DOT1L somatic mutations and predictions of their damaging effects in melanoma.

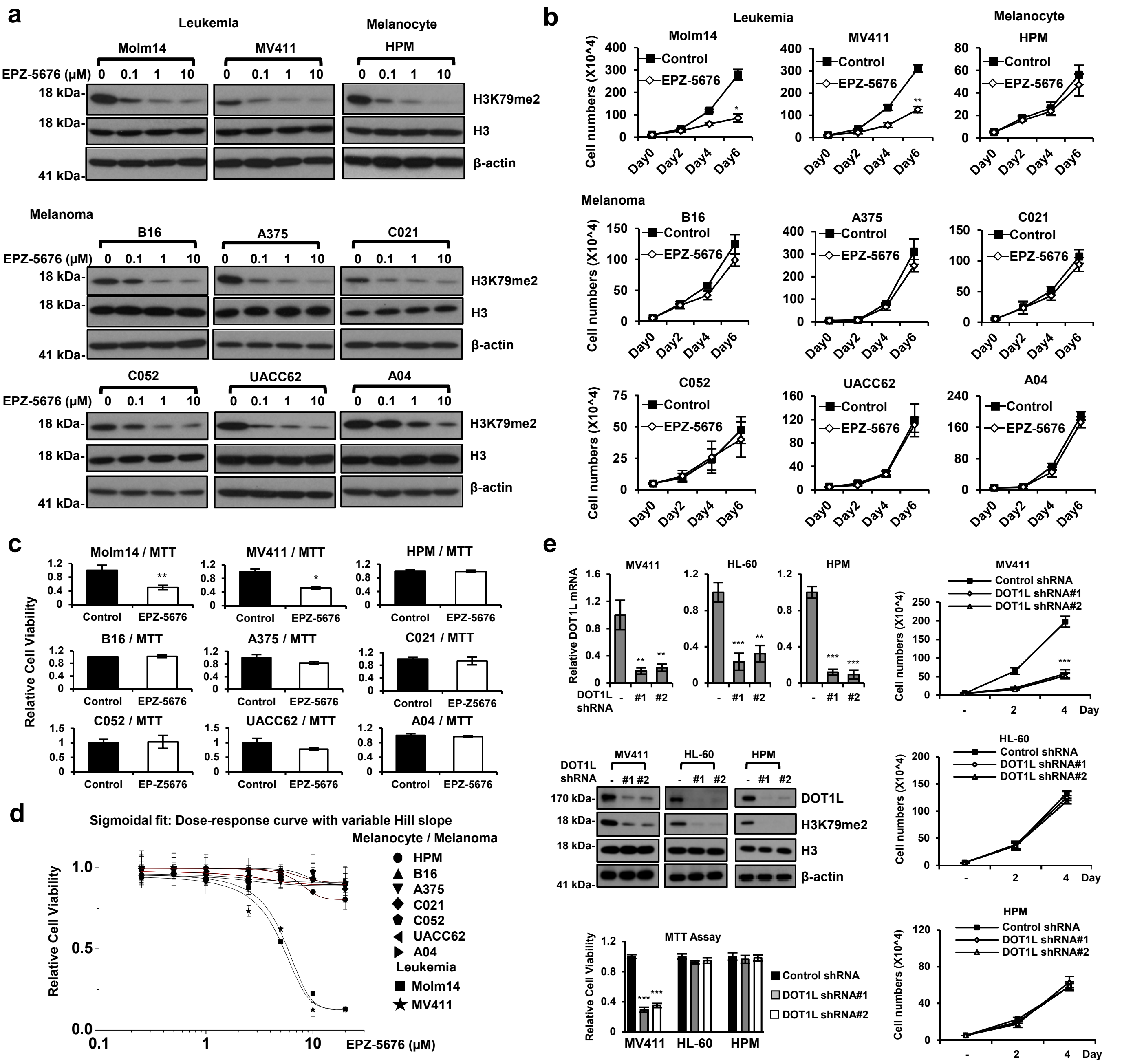

Supplementary Figure 2. DOT1L inhibition suppresses cell proliferation selectively in MLL-rearranged leukemia cells, but not in non-MLL-rearranged leukemia or melanoma.

(a-d) Leukemia cell lines, HPMs and melanoma cell lines as indicated were treated with a DOT1L inhibitor, EPZ-5676 (5μM). Levels of di-methylated H3K79 and H3 proteins **(a)** , cell numbers **(b)** and cell viability **(c-d)** was evaluated. **(e)** HPMs and non-MLL-rearranged leukemia HL-60 cells were infected with shDOT1L to deplete endogenous DOT1L. The expression of DOT1L mRNA were detected by qRT-PCR. The resulting viable cell numbers were counted or measured by MTT assay. Protein expressions of DOT1L, di-methylated H3K79 and H3 were detected by Western blot. *$p < 0.05$, **$p < 0.01$, ***$p < 0.001$, unpaired Student's *t*-test. Error bars represent ± s.d.

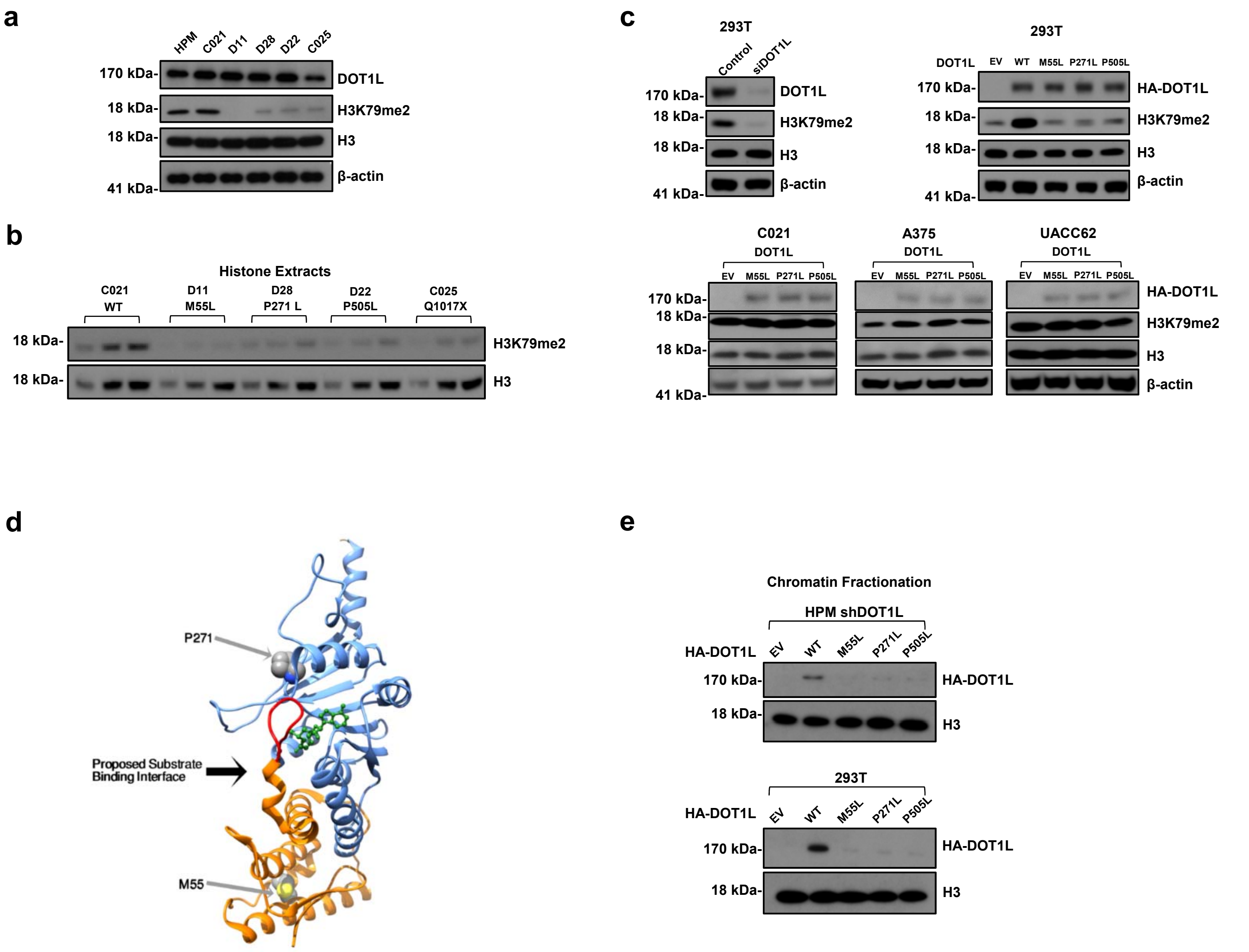

Supplementary Figure 3. DOT1L loss-of-function mutations in human melanoma.
(a) HPM and primary melanoma cells were assayed with Western blot. **(b)** Histone extracts isolated from melanoma cells were assayed with Western blot. **(c)** Control of siDOT1L were introduced into 293T cells (top left panel). WT or Mutant DOT1L constructs were transfected into 293T cells (top right panel). Mutant DOT1L constructs were transfected into C021, A375 or UACC62 cells (bottom panels). **(d)** Locations of M55 and P271 on crystal structure of the N-terminal catalytic domain of hDOT1L (PDB code: 1NW3). The N-terminal region (4-126) is shown in orange. The SAM-binding C terminal region (141-332) is shown in blue. A flexible loop (122-140) between the N-terminal and C-terminal regions is shown in red. Cofactor SAM is show in green stick-and-ball format. The M55 and P271 are shown in space-filled format. **(e)** DOT1L depleted HPMs re-expressed with DOT1L wild type or mutants were used to isolate the chromatin fractionation, which were assayed with Western blot to test mutated DOT1L association on chromatin.

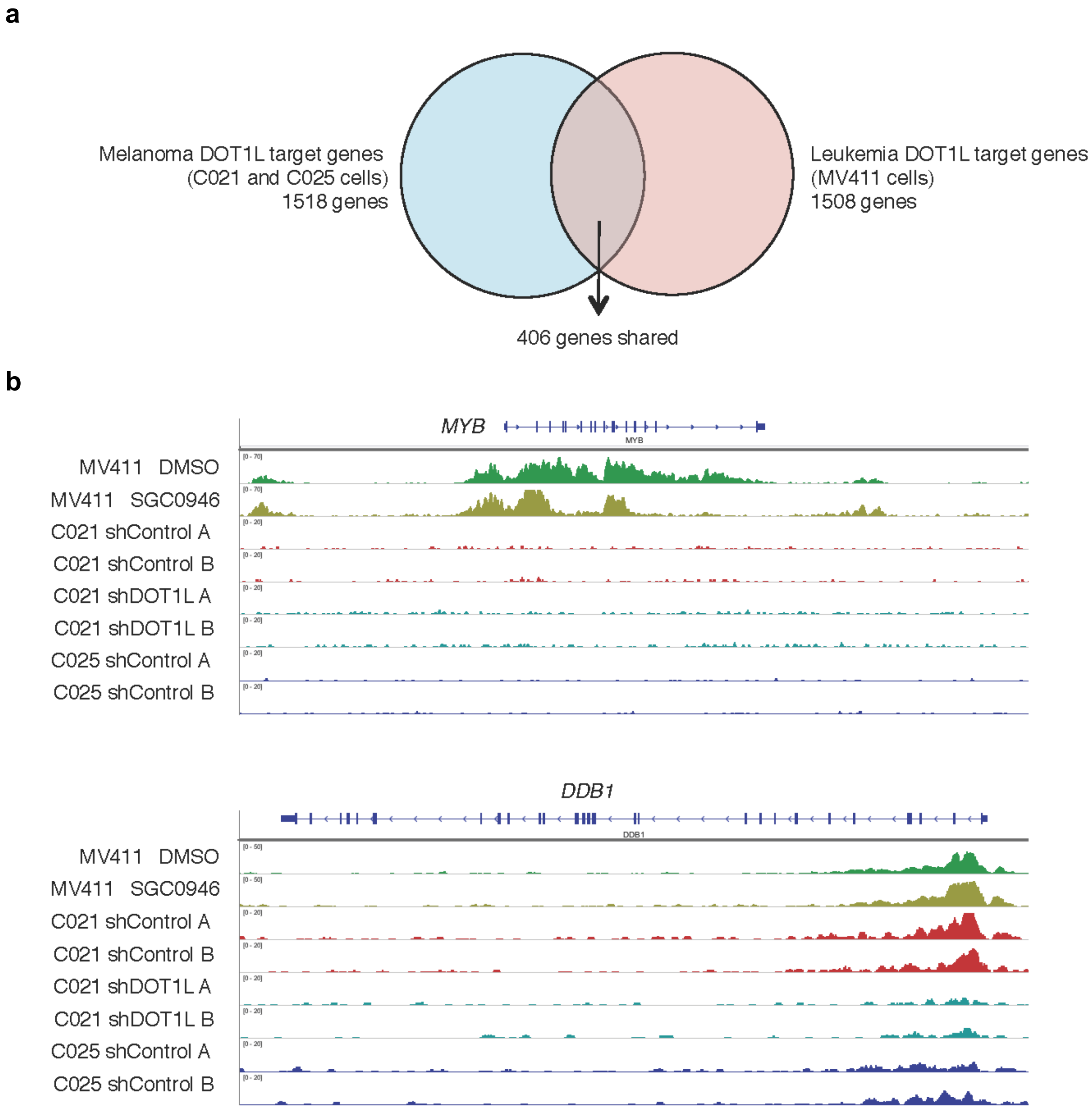

Supplementary Figure 4. Comparison of the ChIP-seq results of DOT1L/H3K79me2 between melanoma and MLL-rearranged leukemia cells.

(a) Numbers of DOT1L/H3K79me2 targeted genes by ChIP-seq in melanoma (C021 and C025) only, Leukemia (MV411) only and shared are displayed using Venn diagram. **(b)** ChIP-seq tracks for represent genes *MYB* and *DDB1*. Number counts for peaks are present on y-axis.

a

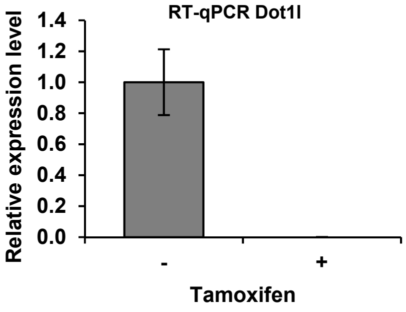

b

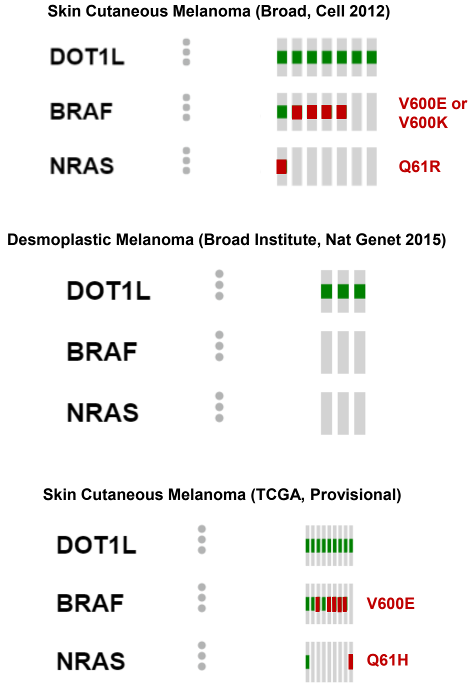

c

Tumor / Mutation	1	2	3	4	5	6	7
Braf V637	+	+	+	-	-	-	+
Nras Q61	-	-	-	-	-	-	-
Nras G12,13	-	-	-	-	-	-	-

Supplementary Figure 5. BRaf mutations in melanoma from DOT1L-null mice.
(a) RT-PCR was performed with isolated melanocytes by flow cytometry from *DOT1L*$^{flox/flox}$*/Tyr-CreERT2* mice administrated with/without tamoxifen as indicated, for detecting knockout efficiency. **(b)** Mutation co-occurrence test between DOT1L mutations and the BRaf and/or NRas mutations in human melanomas collected in cBioPortal. Green bar represents gene mutation, and red bar represents the specific mutation indicated. **(c)** BRaf V637 mutation and/or NRas Q61, G12, 13 mutations in melanomas from DOT1L-null mice.

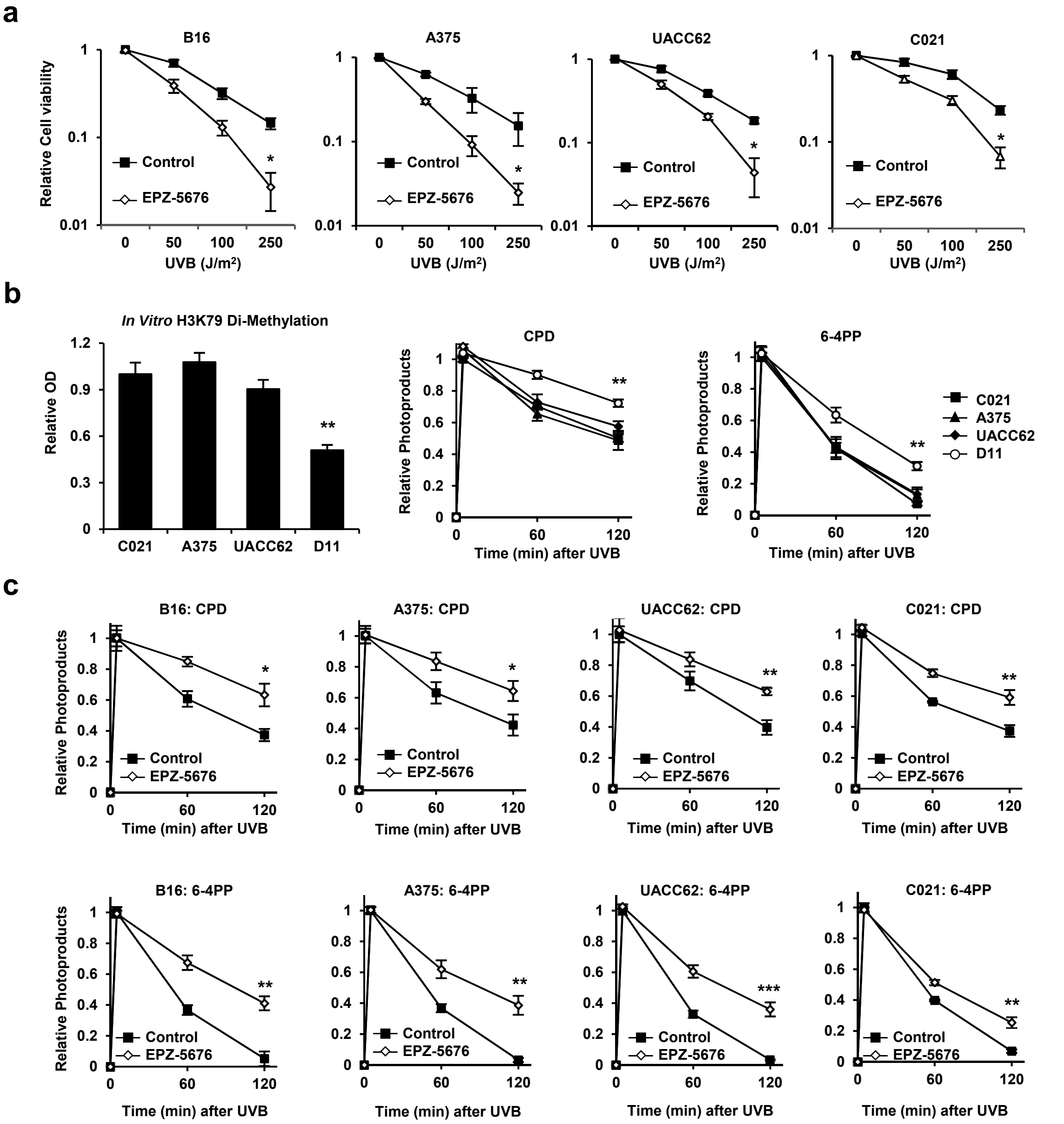

Supplementary Figure 6. DOT1L is crucial in UV-induced DNA damage repair.

(a) Melanoma cells as indicated were treated with EPZ-5676 (5 μM) and then irradiated with different doses of UVB as indicated. Cell viability was measured by MTT assay 24 h after UVB irradiation. **(b)** Melanoma cells as indicated were irradiated with 100 J m^{-2} UVB. Histones were extracted from melanoma cells to measure H3K79me2 by ELISA. Genomic DNA was extracted. Photoproducts were detected by ELISA with a pyrimidine dimer CPD specific antibody or a 6-4PPs-specific antibody. **(c)** Melanoma cells as indicated were irradiated with 100 J m^{-2} UVB and treated with EPZ-5676 (5 μM) for 24 hours post irradiation. Genomic DNA was extracted. Photoproducts were detected by ELISA with a pyrimidine dimer CPD specific antibody or a 6-4PPs-specific antibody. *$p < 0.05$, **$p < 0.01$, ***$p < 0.001$, unpaired Student's *t*-test. Error bars represent ± s.d.

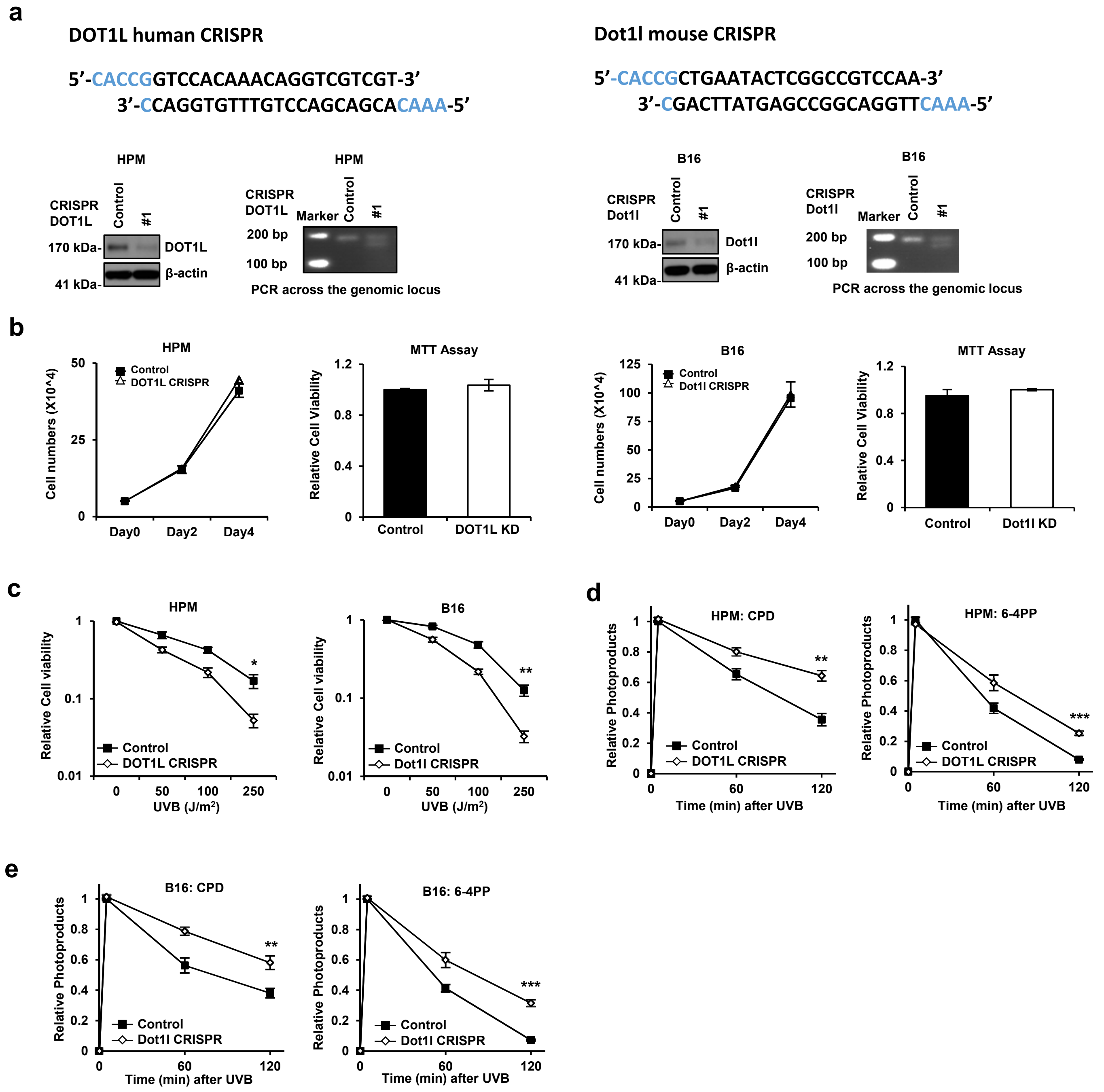

Supplementary Figure 7. DOT1L knockdown with CRISPR reduces UV-induced DNA damage repair.
(a) List of primers which were used to construct CRISPR induced DOT1L deletion in HPMs and B16 cells. Western Blot and PCR across the genomic locus were used to verify the effect of CRISPR. **(b)** Viability of control and DOT1L CRISPR cells were measured by cell number counting or MTT assay as indicated. **(c)** Control and DOT1L CRISPR cells as indicated were irradiated with different doses of UVB as indicated and then cell viability was measured by MTT assay 24 h after UVB irradiation. **(d-e)** Control and DOT1L CRISPR cells were irradiated with 100 J m^{-2} UVB. Genomic DNA was extracted. Photoproducts were detected by ELISA with a pyrimidine dimer CPD specific antibody or a 6-4PPs-specific antibody. *$p < 0.05$, **$p < 0.01$, ***$p < 0.001$, unpaired Student's *t*-test. Error bars represent ± s.d.

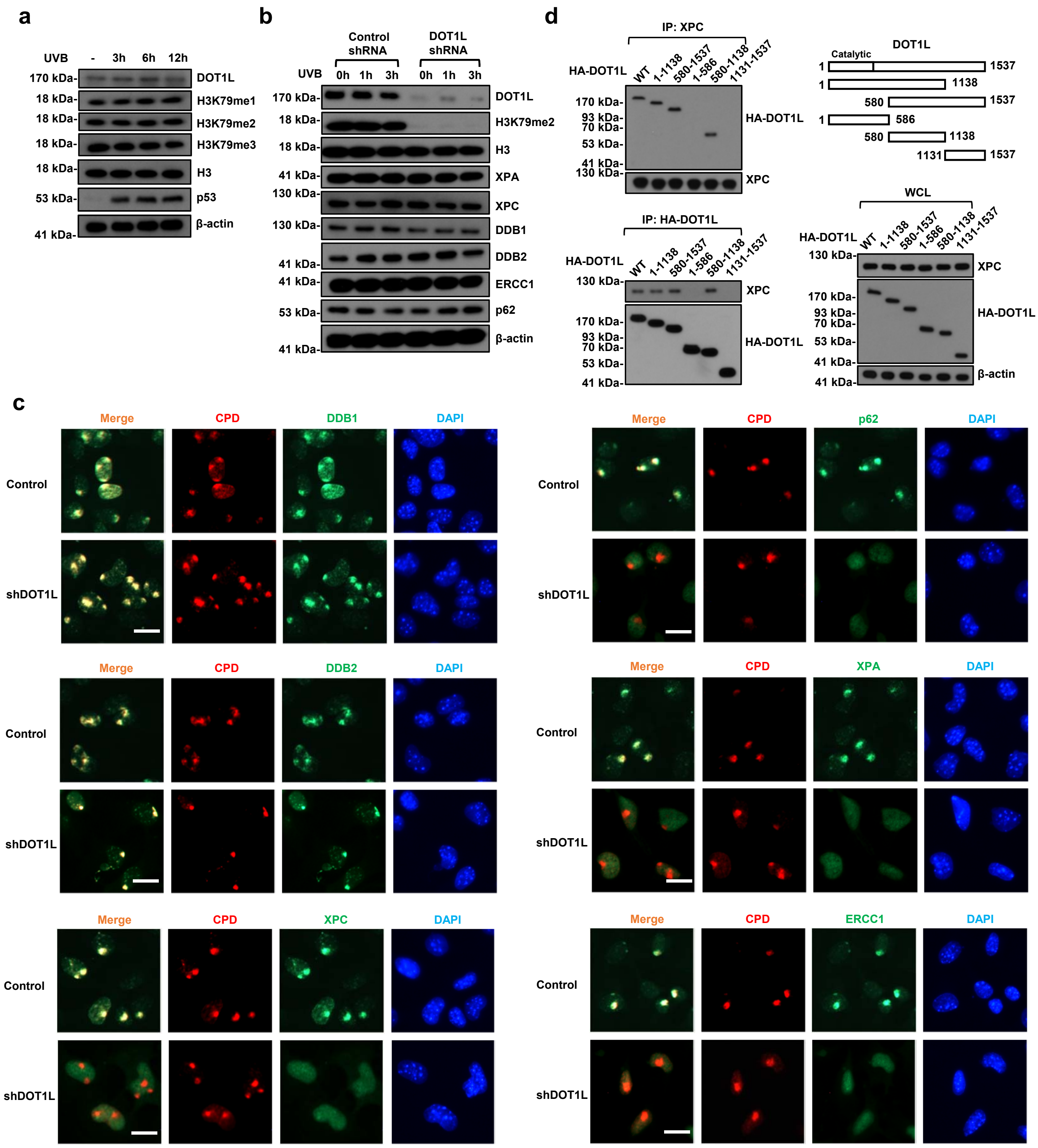

Supplementary Figure 8. DOT1L interaction with XPC is required for NER repair on UVB-induced DNA damage. **(a-b)** Protein expressions in the NER signaling pathway after UVB irradiation in melanocytes. HPMs or HPMs with stable shDOT1L were irradiated with 100J m^{-2} UVB, and then harvested at different time points as indicated to perform Western blot using the indicated antibodies. **(c)** HPM cells with DOT1L depletion or shControl were subjected to 100 J m^{-2} UVB under 5 μm micropore filter and after 0.5 hour were co-stained for CPD with DDB1, DDB2, XPC, p62, XPA or ERCC1. DAPI, 4',6-diamidino-2-phenylindole. Scale bar, 20 μm. (**d**) B16 cells were transfected with various HA-DOT1L constructs together with XPC. After 48 hours, the cells were treated by irradiation with 100 J/m^2 UVB before harvesting for immunoprecipitation (IP) and immunoblot (IB) analysis.

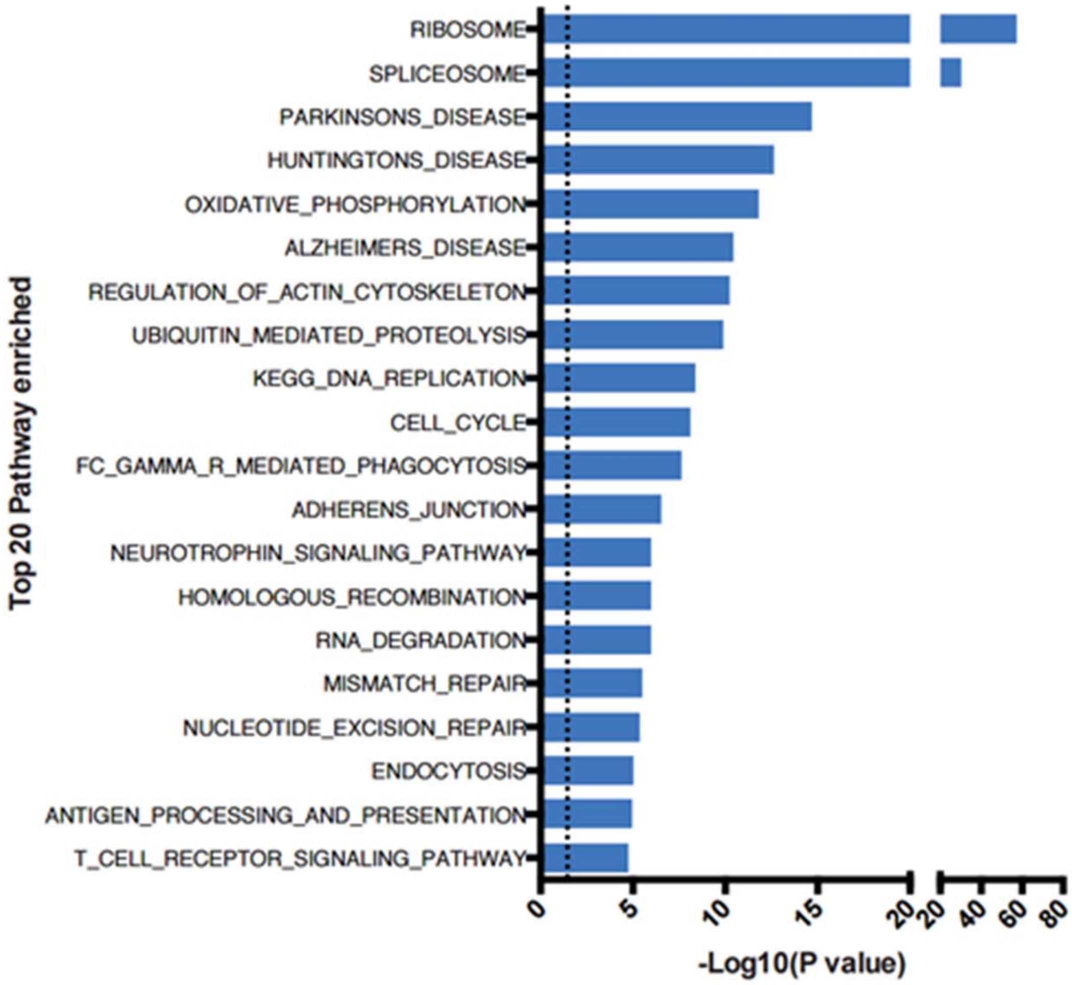

Supplementary Figure 9. Enriched DOT1L-targeting pathways.
Pathway analysis on ChIP-seq data using the KEGG dataset.

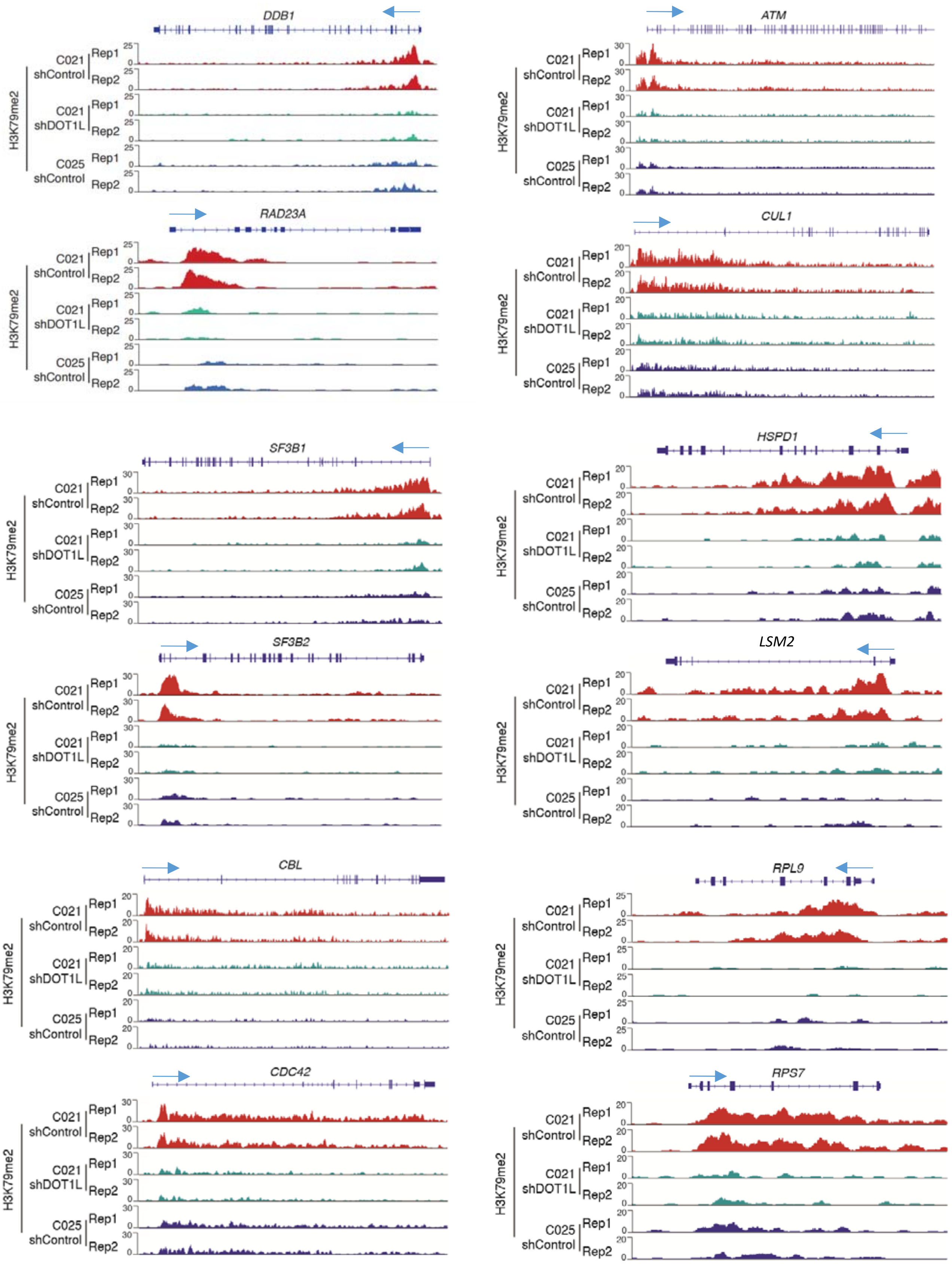

Supplementary Figure 10. Example DOT1L/H3K79me2 regulated genes identified by ChIP-seq.

Figure 2

b

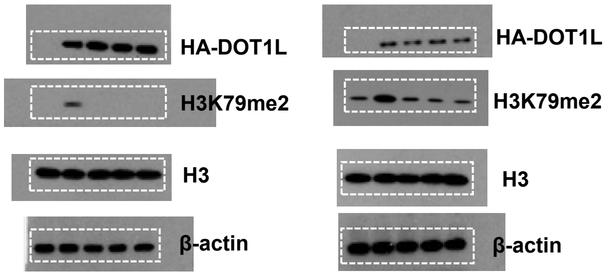

Figure 3

a

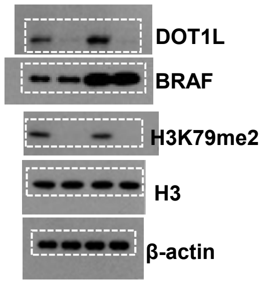

d

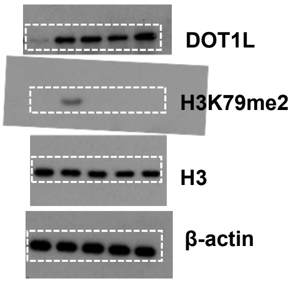

Figure 5

i

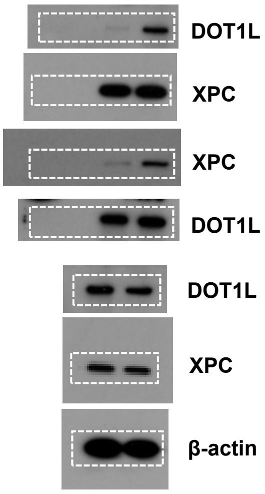

j

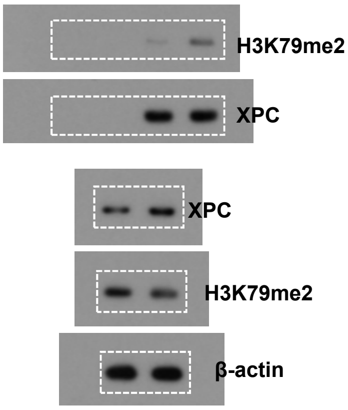

k

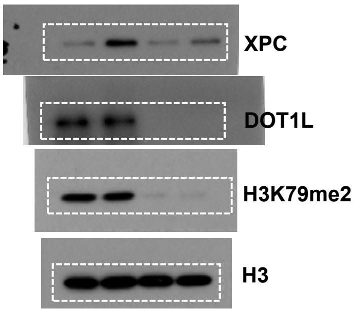

Supplementary Figure 11. Uncropped scans of the most important Western blots.
