# Supplementary material for: The protective role of DOT1L in UV-induced melanomagenesis
Source: Nat Commun. 2018 Jan 17;9:259. doi: 10.1038/s41467-017-02687-7 (PMC5772495; doi:10.1038/s41467-017-02687-7)
Supplement: Supplementary file 2 — Description of Additional Supplementary Files [file 41467_2017_2687_MOESM2_ESM.pdf]

Description of Additional Supplementary Files

File Name: Supplementary Data 1
Description: Mutation of DOT1L in patients.

File Name: Supplementary Data 2
Description: Annotation of somatic variants in DOT1L.

File Name: Supplementary Data 3
Description: Summary of DOT1L mutations.

File Name: Supplementary Data 4
Description: Sequencing of human melanoma cell lines.

File Name: Supplementary Data 5
Description: KEGG pathways analysis of ChIP-seq results.

File Name: Supplementary Data 6
Description: Microarray analysis of NER genes.

File Name: Supplementary Data 7
Description: KEGG pathways analysis of microarray results.
